# Mathematical modeling of public health policy for international travelers (PHPIT): a case study of the COVID-19 pandemic in Thailand

**DOI:** 10.1186/s12889-025-25811-5

**Published:** 2025-12-01

**Authors:** Vidhyakorn Mahd-Adam, Chawarat Rotejanaprasert, Saranath Lawpoolsri, Panithee Thammawijaya, Wirichada Pan-ngum

**Affiliations:** 1 Department of Tropical Hygiene, Faculty of Tropical Medicine, Mahidol University, Bangkok, 10400 Thailand; 2 Mahidol-Oxford Tropical Medicine Research Unit (MORU), Faculty of Tropical Medicine, Mahidol University, Bangkok, 10400 Thailand; 3 Division of Epidemiology, Department of Disease Control, Ministry of Public Health, Nonthaburi, 11000 Thailand

**Keywords:** Mathematical model, Public health policy, COVID-19, International travelers, Air travel, Thailand, Pre-implementation evaluation, Test-and-go

## Abstract

**Background:**

Following border closures implemented during the COVID-19 pandemic, Thailand subsequently reopened its international borders, employing public health policy for international travelers (PHPIT) to mitigate the importation of COVID-19 cases and minimize potential missed cases. These measures included departure country risk classification, vaccination certificate requirement, pre-departure and entry testing, and quarantine.

**Methods:**

To assess the effectiveness of these interventions, we developed a cross-border control model, which integrates cluster analysis and mathematical modeling to estimate reductions in missed cases in Thailand under various scenarios.

**Results:**

Our findings indicated that, as a single measure, when compared with implementing no interventions, quarantine for 1, 3, 5, 7, 10, and 14 days reduced missed cases by 45%, 58%, 72%, 80%, 88%, and 94%, respectively. Departure country risk classification reduced missed cases by up to 72% and vaccination certificate requirement by up to 24%. Testing once at 72, 48, 24, or 0 h pre-departure reduced missed cases by 5%, 7%, 10%, and 14% respectively. Entry testing (the “test-and-go” policy) reduced missed cases by 29%. Strategically combining quarantine with other PHPIT measures could achieve a 46–98% reduction in missed cases, with the maximum reduction in missed cases when implementing 14-day quarantine together with pre-departure testing.

**Conclusions:**

Quarantine could serve as a standard measure to minimize missed cases and be optimized by a vaccination certificate requirement. Meanwhile, testing and departure country risk classification could be withdrawn due to their minimal effectiveness and potential for discrimination, respectively. The combinations and their optimization should be guided by domestic transmission dynamics, healthcare system capacities, societal perspectives, and economic adaptability. Future PHPIT implementation should incorporate pre-evaluation to balance intervention effectiveness, equity for international travelers, and public health and economic security.

**Supplementary Information:**

The online version contains supplementary material available at 10.1186/s12889-025-25811-5.

## Background

The COVID-19 pandemic had a major impact on global travel. Thailand initiated public health interventions on 3 January 2020 and detected its first air travel-related infection on 13 January 2020 [1]. Ultimately, all international arrival passengers were required to have a medical certificate and health insurance [2–4]; this was followed by an international flight ban from April 2020 to October 2021 [4]. The border closures led to a 90% decrease in travelers, from 39.7 million in 2019 to 3.8 million in 2020, causing Thailand’s GDP to drop by 6.1% [5].

To address economic concerns while managing public health risks, Thailand implemented a four-period border reopening strategy [6], see Table S2. Period A required a 14-day quarantine period with restrictions on movement. Period B reduced quarantine to 10 days with limited travel and a requirement to stay in designated facilities. Period C, implemented for travelers from countries that had reached 70% vaccine coverage, either did not require quarantine or required a one-day quarantine while awaiting a test result upon arrival. Period D involved no quarantine for the same target countries as in the third period. In all periods, a vaccine certificate and testing 72 h pre-departure were required.

Despite these measures, imported COVID-19 cases continued to occur (Table S1). From April 2020 to April 2022, Thailand screened 2 million international arrivals, detecting approximately 20,000 imported cases (1.2% of arrivals) [7, 8]. The imported cases largely comprised returning Thai citizens (Table S3, Figure S8) [8, 9] and were reported to the Department of Disease Control to inform management strategies and policy to mitigate domestic transmission. However, the less-than-perfect effectiveness of public health policy for international travelers (PHPIT) measures meant many cases were not identified and therefore not under management or control. This led to underrepresentation of the total number of imported cases, which impacted local transmission and the administration of control strategies. Although these measures were initially successful in curtailing case importation, their long-term sustainability and effectiveness in diverse scenarios remain underexplored, underscoring the need for a systematic retrospective evaluation of PHPIT strategies.

We conducted a mathematical modeling study to retrospectively evaluate the effectiveness of PHPIT in reducing missed cases under various scenarios. Local and global COVID-19 data were used in developing the model, which was based on the actual PHPIT implemented in Thailand. We conducted a cluster analysis of other countries and estimated the number of missed cases. Here, we present an analysis of the various PHPIT measures, to inform future policymaking.

## Methods

### Six stages of PHPIT implemented in Thailand during the COVID-19 pandemic

Based on the procedures required for international travelers to enter Thailand during the COVID-19 pandemic (Figure S1 [10]), we categorized the diverse interventions, infection risks, disease progression, and waiting times encountered throughout travel into six distinct stages (Table S5).

#### Stage P1 - vaccination certificate required

Travelers to Thailand required a certificate of entry (three days for initial approval). They then had 15 days to submit their vaccination certificate, followed by a 3-day wait for final approval. We assumed some travelers might have been exposed to the virus during this stage. The impact of the vaccination certificate requirement carried through to all subsequent stages.

#### Stage P2 - waiting period before testing

If pre-departure testing was required for inbound travelers to Thailand, individuals positive for COVID-19 would be denied permission to travel at this stage. Testing timelines varied based on Thailand’s PHPIT requirements.

#### Stage P3 - period following testing

Travelers testing negative were able to travel to Thailand. Although testing reduces the number of potentially infectious individuals, the proportion of infected travelers increases over time due to ongoing exposure risks in the local country.

#### Stage P4 - travel to Thailand

Travelers proceeded with their trips, encountering varying risks of infection. Infected individuals who developed severe symptoms during stages P1 to P3 were assumed to cancel their trip. During air travel, infection risks vary due to factors such as altered contact patterns, traveler density in airports and on aircraft, and potential lapses in hygiene practices.

#### Stage A - arrival in Thailand

Travelers not subject to quarantine were allowed unrestricted entry to Thailand. However, individuals exposed to the virus during the prior stages could potentially spread the infection within local communities. Interventions in this stage included testing on entry, screening for symptoms, and certificate of entry (COE) validation.

#### Stage Q - quarantine measures

If required, travelers were directed to pre-booked quarantine facilities upon arrival. We assumed no new infections occurred during the quarantine period. Before release, travelers underwent testing to mitigate contagion.

To analyze these processes, we integrated the six stages (P1–Q) of PHPIT, incorporating the transmission dynamics of COVID-19 via an SEIR (susceptible, exposed, infected, recovered) compartmental model and the departure country risk classification across the four periods of border reopening. This conceptual framework formed the basis of our cross-border control (CBC) model, designed to evaluate the impacts of PHPIT on Thailand’s border reopening strategies.

## Model development

### Cross-border control model

**Fig. 1:**
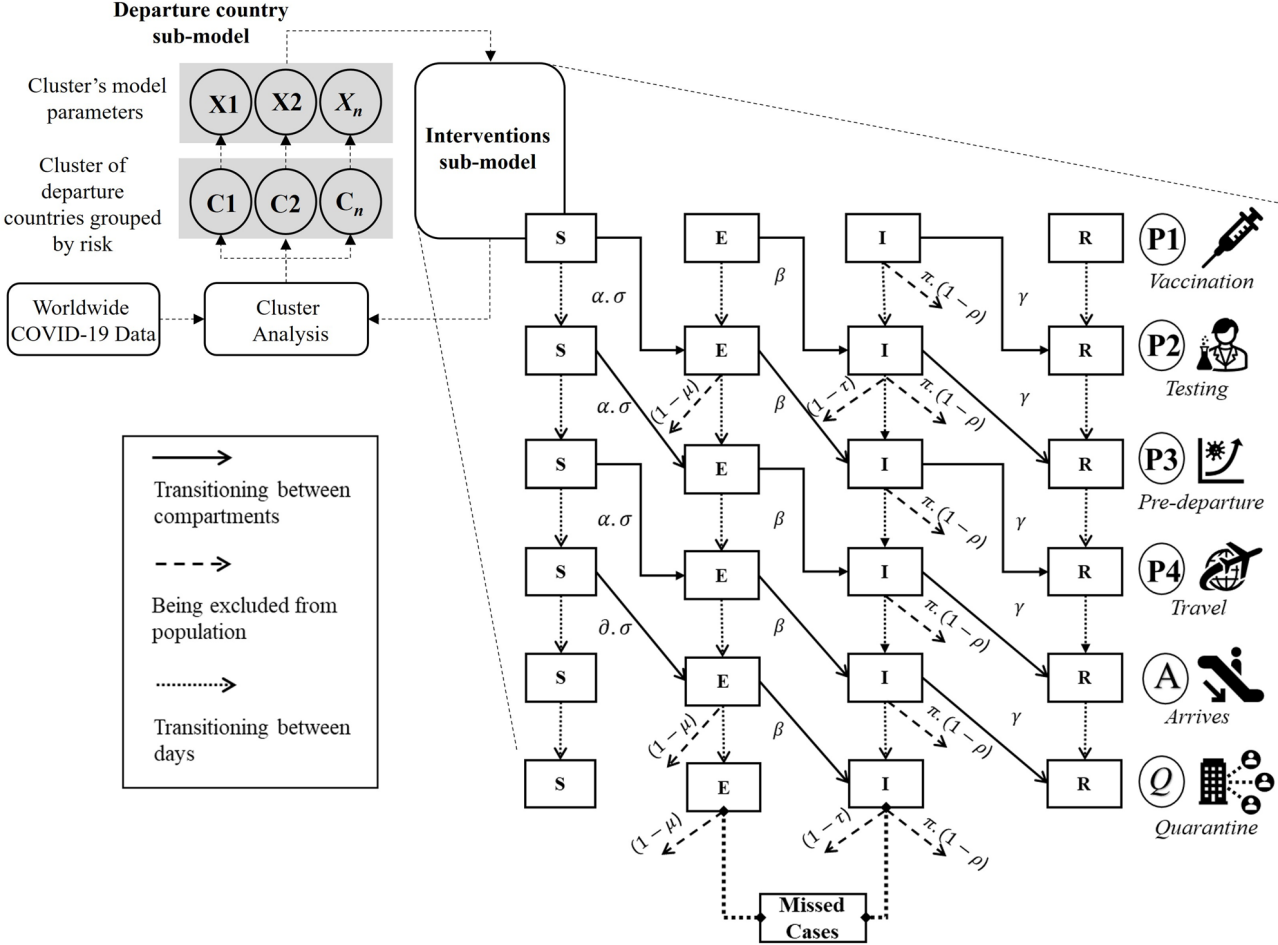
The cross-border control (CBC) model included a departure country sub-model (top left), incorporating cluster analysis to generate clusters of departure countries grouped by risk (C1, C2, …, C_n_) to compute average values of infection rate, testing positivity, and vaccine coverage for each cluster for the model’s parameters (X1, X2, …, X_n_). The CBC model also included an intervention sub-model (right side) comprising the six stages of PHPIT (P1, vaccination; P2, testing; P3, after testing; P4, travel; A, arrival; Q, quarantine). This was integrated with an SEIR (susceptible, exposed, infectious, recovered) compartmental model to simulate the transmission dynamics of the COVID-19 cases and calculate the number of missed cases. Duration between stage P1 (vaccination) to P2 (testing) was 2–5 days, stage P2 (testing) to P3 was 0–3 days, stage P3 to P4 (travel) was 1 day, stage P4 (travel) to A (arrival) was 1 day, and stage A (arrival) to Q (quarantine) was 0–14 days. ($\:\alpha\:$ = infection rate before traveling; $\:\partial\:$ = infection rate during the period of travel; $\:\sigma\:$ = vaccine-associated reduction in infection rate; $\:\beta\:$ = rate of being infectious; $\:\gamma\:$ = recovery rate; $\:\pi\:$ = proportion of symptomatic infected individuals; $\:\rho\:$ = vaccine-associated symptom reduction; $\:\mu\:$ = false-negative test result during the latency period; $\:\tau\:$ = false-negative test results during the infectious period)

The CBC model comprised two distinct components (Fig. 1). The first component, which is beyond direct control, involves vaccination coverage and infection rates influenced by control measures and policies in each traveler’s country of departure. To evaluate the impacts arising from this component, we constructed the departure country sub-model, which utilizes cluster analysis (Fig. 1, top left) to classify departure countries by risk. This approach generates clusters of departure countries grouped by risk (C) and is followed by calculating the model’s parameter (X) for each cluster. The second component focuses on directly implemented interventions to reduce the risk of case importation and transmission. To assess the effectiveness of these interventions, we developed the intervention sub-model (Fig. 1, bottom right).

### Departure country risk classification (departure country sub-model)

We used K-means cluster analysis and principal component analysis [11]. Optimal numbers of clusters (K) were determined using three methods: elbow, silhouette, and the gap statistic. K-means cluster analysis was applied to classify all departure countries based on three input variables: (i) infection rate, (ii) test positivity values, and (iii) vaccination coverage data. Departure countries were grouped into clusters to represent the risk of transmission in each country. Chi-square tests were performed to compare the clusters of departure countries grouped by risk.

### Modeling of COVID-19 transmission dynamics (interventions sub-model)

The interventions sub-model was created by adjusting the transmission dynamics of COVID-19 disease to account for the six stages of cross-border travel outlined in the PHPIT. Each stage exhibits unique functional behaviors, parameters, and time durations, which are dependent on the interventions implemented. The timeframes between stages (P1–P4 to A–Q) are influenced by the duration of the specific intervention applied. We developed a deterministic SEIR compartmental model [12] to incorporate the spatial-temporal transmission dynamics of COVID-19 across the six stages of travel.

We delineated spaces where COVID-19 transmission could occur, covering the period of travel from vaccination to quarantine. Transmission dynamically progressed through these stages, with COVID-19-free individuals in the susceptible (S) traveler compartment transitioning to the exposed (E) compartment at the infection rate before traveling $\:\left(\alpha\:\right)$ and during the period of travel $\:(\partial\:)$. There was a vaccine-associated reduction $\:\left(\sigma\:\right)\:$in this infection rate. Exposed individuals then became infectious and transitioned to the infectious (I) compartment at the infectious rate $\:(\beta\:)$, eventually moving to the recovered (R) compartment at the recovery rate $\:\left(\gamma\:\right)$. Presymptomatic transmission accounts for a mean of 58% (range: 37–80%) of COVID-19 transmission across locations [13–16], and we adopted a baseline 50% to 50% initial infection distribution between exposed (presymptomatic infection) and infectious (symptomatic infection) compartments.

Before traveling to Thailand (stages P1–P3), a proportion of symptomatic infected individuals $\:\left(\pi\:\right)$ who had not experienced vaccine-associated symptom reduction $\:(1-\rho\:)$ were assumed to discontinue travel due to severe symptoms, while after arrival (stages P4, A, and Q), severely symptomatic travelers could be detected in quarantine. Additionally, in P2, A, and Q, testing requirements meant some exposed individuals were excluded from traveling at a rate of $\:(\:1-\:\mu\:)$, where $\:\left(\mu\:\right)$ is a false-negative test result during the latency period with a rate of $\:(1-\tau\:)$, where $\:\left(\tau\:\right)$ accounts for false-negative test results during the infectious period. Numbers of “missed cases” comprise the cumulative number of exposed and infectious individuals entering the community. The model equations, parameter values, and definitions are provided in Tables S4 and S5.

In this model, all quarantined travelers underwent exit testing prior to release to mitigate community transmission. Given the possibility of false-negative results, infected individuals could remain undetected and enter the community, potentially contributing to domestic COVID-19 transmission. These undetected infected individuals (“missed cases”) are a key model output.

### Data sources

We used data on total monthly travelers and total reported imported cases (April 2020–April 2022) [7, 17]. For the model simulation, we employed daily data on infection rates, testing positivity, and vaccination coverage from 260 countries between 1 January 2020 to–9 January 2022 [18]. The data were processed into seven-day average values.

### Model simulation

We used Microsoft Excel [19] to create calculation and dashboard sheets for the SEIR discretized model and simulation (Figures S2 and S3). The dashboard included parameter values, scenario selection, and a results table predicting the number of missed cases before and after quarantine. The model was used to simulate a single event over 7 to 22 days, tracking cumulative missed cases from day 8 onward based on various scenarios and interventions. Each column represented the number of individuals in each SEIR state on the day. The cluster analysis was performed using the R programming language [20].

### Sensitivity analysis

To assess the impact of value variations on model outcomes, we performed a one-way sensitivity analysis by varying the 5% false-positive rate [21] in the susceptible and recovered compartments and the initial number of travelers (2019 cluster-specific historical numbers versus 100,000 per cluster). Based on the 50%–50% baseline initial infection distribution between exposed and infectious compartments previously mentioned, sensitivity analysis spanning 50% points (25% to 75%; 75% to 25%) between the two compartments was also performed.

### Model validation

For clustering analysis, internal validation included average silhouette width (ASW), the Calinski-Harabasz index (CHI), and the Dunn index (DI). Algorithmic consensus was assessed by comparing K-means with Ward’s hierarchical clustering method and partitioning around medoids (PAM) via adjusted Rand index (ARI). All analyses used standardized data.

We validated the intervention sub-model by comparing its estimated imported cases with reported imported cases under comparable PHPIT scenarios. The model-derived estimates were calculated based on infected individuals detected after arrival in Thailand, including cases identified through entry testing, quarantine testing, and symptomatic presentation. The officially reported cases (April 2020–April 2022) [17] were selected from 26 risk type as imported cases, type then categorized into4 groups (Table S14) and were adjusted with numbers of travelers [7] to match the model’s weekly rate of 100,000 travelers for the same PHPIT scenarios (Tables S8, S15-A, and S15-B). For temporal comparison, monthly trends were separated into four periods (Period A–D) corresponding to major PHPIT policy changes (Table S2). Model outputs (mean and range) were visualized and evaluated using the adjusted reported imported cases.

### Model assumptions

Our model incorporated various assumptions based on COVID-19 epidemiology and transmission. We assumed that a fixed number of 100,000 travelers each week registered for a COE before arriving in Thailand. The simulation was based on a fixed number of travelers per week. We assumed vaccines conferred partial protection, reducing vaccinated individuals’ susceptibility by 30% and severity of symptoms by 70% [22, 23]. We focused exclusively on air travel, excluding ground and sea travel due to distinct characteristics and associated risks [24]. Infection and transmission risks were assessed throughout the entire cross-border travel process without incorporating the force of infection, due to the characteristics of the population. We also assumed a constant, uniform symptomatic proportion, which overlooks presymptomatic delays and symptom progression dynamics, compromising the model’s accuracy in stage-specific case identification. The model’s population was evaluated at the cluster level, although individuals within a cluster do not necessarily reside in the same community and do not influence each other’s infection risk. We assumed that infection risk during travel was twice as high as in a traveler’s local country, given the pathogen’s likely advantage in settings involving intense close contact, e.g., public transport, airports, and aircraft [25, 26]. Variant transmissibility impacts were captured via our infection rate parameter, derived from real-time seven-day cumulative infection data. The impacts of interventions were assessed for regular border crossings but excluded illegal entry, which involves unique risks. Symptom screening was not incorporated. Quarantining travelers stayed in separate rooms under strict infection control measures, including multiple RT-PCR tests, daily symptom monitoring, physical distancing, plus contact tracing if cases in quarantine were confirmed, with high-risk contacts mandated to undergo extended quarantine and testing [27]. Hence, for simplicity, transmission during quarantine was excluded from this model.

### Scenarios of interest

**Fig. 2:**
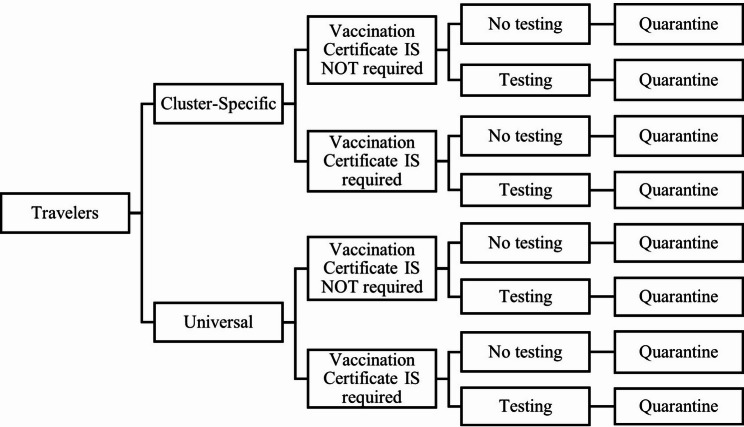
Decision tree of model scenarios, including departure country risk classification (cluster-specific and universal measures), vaccination certificate requirement (vaccination certificate is required means all travelers are vaccinated; vaccination certificate is not required means travelers may have any vaccine status), testing (no testing; testing at 0, 24, 48, or 72 h pre-departure; and testing on entry), and quarantine measures (0, 1, 3, 5, 7, 10, or 14 days)

We created 336 simulation scenarios encompassing four PHPIT categories: (i) departure country risk classification, including both cluster-specific and universal (non-discriminatory) measures; (ii) vaccination certificate requirement (required/not required); (iii) testing strategy – options included no testing, pre-departure testing at 0, 24, 48, or 72 h, or testing on entry; and (iv) quarantine duration of 0, 1, 3, 5, 7, 10, or 14 days (Fig. 2).

## Results

### Risk classification of departure countries

Classification of departure country with two principal components (PC) explained 87.2% of total variance. PC1 (explaining 53.2% of the variance) was predominantly influenced by infection rate (50.61%) and testing positivity rate (47.56%), representing transmission risk and probability, respectively. PC2 (explaining 34% of the variance) was driven by vaccination coverage (93.51%), reflecting population-level immunity (Figure S4). We identified three clusters of departure countries (Figures S5–S6). Cluster 1 (high PC1, low PC2) exhibited a high level of COVID-19 spread and active testing but low vaccine uptake, while cluster 2 (low PC1, high PC2) exhibited good vaccine coverage that suppressed infections, possibly with less testing needed, and Cluster 3 (medium PC1, medium PC2) exhibited balanced transmission and detection, with partial vaccine protection. The analysis included 111 countries with complete daily data on infection rates, test positivity, and vaccination coverage for seven consecutive days (retrospectively from 1 to 9 January 2022). These were distributed across clusters as follows: Cluster 1 (*n* = 47), Cluster 2 (*n* = 20), and Cluster 3 (*n* = 44) (see Table S6 for the complete list). The mean infection rates (per million) for clusters 1, 2, 3, and universal measures applied to travelers from all countries were 142.09, 650.58, 48.72, and 196.70, respectively (Table 1). Regarding vaccination coverage, the mean values for clusters 1, 2, 3, and universal measures were 65.81%, 40.90%, 19.15%, and 42.83%, respectively. The probabilities of testing positive were 0.07, 0.05, 0.20, and 0.05 for clusters 1, 2, 3, and universal measures, respectively. When comparing the universal measures with clusters 1, 2, and 3, no significant differences were observed in infection rates (*P* = 0.967, 0.876, and 0.925, respectively) or testing positivity (*P* = 0.931, 0.799, and 0.944, respectively). However, vaccination coverage showed statistically significant differences in clusters 1 and 3 compared with universal measures (*P* = 0.001 and *P* < 0.001, respectively).

**Table 1:** Per million mean COVID-19 infection rate, probability of testing positive, and % vaccinated population for each cluster of departure countries grouped by risk and *P*-values among the clusters (*P < 0.01, **P < 0.001)

	Per million mean infection rate	Probability of testing positive	% Vaccinated population
	(*P*-value)	(*P*-value)	(*P*-value)
Universal (***n*** = 111)	**196.70 ** **(ref)**	**0.07** ** (ref)**	**42.82 ** **(ref)**
Cluster 1 (***n*** = 47)	**142.10**	**0.05**	65.81 *
	(0.967)	(0.931)	(0.001)
Cluster 2 (***n*** = 20)	**650.58**	**0.20**	**40.90**
	(0.876)	(0.799)	(0.783)
Cluster 3 (***n*** = 44)	**48.72**	**0.05**	19.15 **
	(0.925)	(0.094)	(< 0.001)

### Estimation of reductions in missed cases

**Fig. 3:**
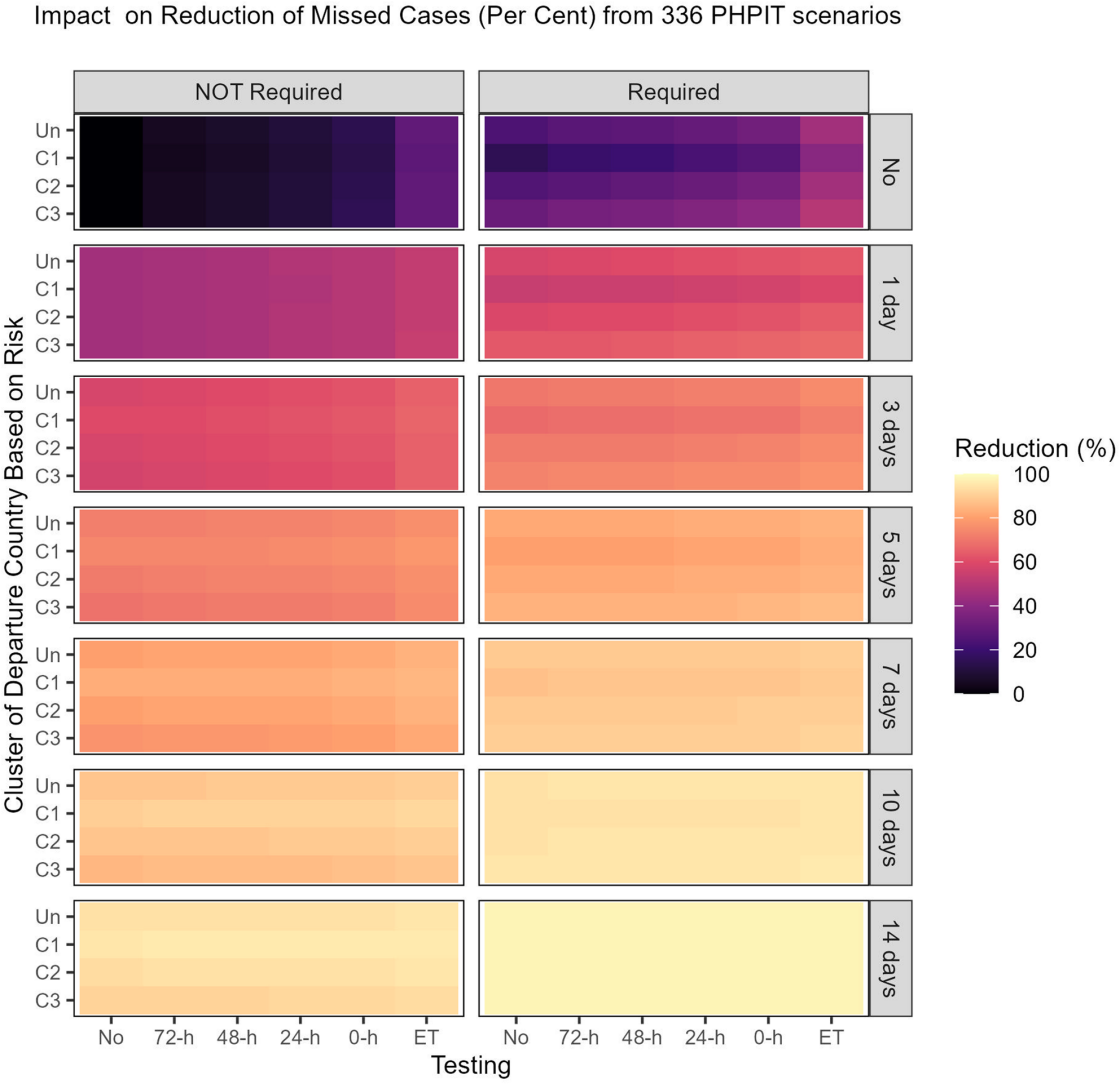
Percentage reductions in numbers of missed cases per week in 336 scenarios compared with the baseline scenarios of taking no action (indicated by * in the table). h, hours pre-departure (0 h refers to immediate testing prior to a flight); ET, entry testing; not required, vaccination certificate is not required; required, vaccination certificate is required

When considering each intervention independently and comparing them with the baseline scenarios with no interventions, requiring a vaccination certificate achieved a 24% reduction in missed cases (Fig. 3, Tables S7 and S9). Testing conducted at 72, 48, 24, or 0 h pre-departure reduced missed cases by 5%, 7%, 10%, and 14%, respectively. Meanwhile, testing on entry resulted in a 29% reduction in missed cases. Quarantine measures of 1, 3, 5, 7, 10, or 14 days reduced missed cases by 45%, 58%, 72%, 80%, 88%, and 94%, respectively.

In the 336 scenario combinations, reductions in missed cases ranged from 4 to 98%; quarantine contributed to reductions of 24–95% compared with no quarantine for the same scenarios. In scenarios where quarantine was implemented for any duration, with pre-departure testing at ≤ 72 h, requiring a vaccination certificate contributed to reductions in missed cases of 2–18%, compared with not requiring a vaccination certificate for the same scenarios, across all departure country risk classifications. However, departure country risk classification itself only contributed to reductions in missed cases of up to 5%, regardless of vaccination certificate requirement. Similarly, pre-departure testing only contributed to reductions in missed cases of up to 5% across all clusters of departure countries based on risk, irrespective of vaccination certificate requirements, while testing on entry contributed up to 9%. Testing on entry without quarantine (the “test-and-go” policy) contributed to a 28–50% reduction in missed cases across all departure country risk classifications and vaccination certificate requirements. Regarding departure country risk classification, across all scenario combinations, universal measures gave the greatest reductions in 26 (17%) combinations, while clusters 1, 2, and 3 gave the greatest reductions in 47 (31%), 27 (18%), and 54 (35%) combinations, respectively; 17 (11%) combinations had the same percentage reduction as departure country risk classification.

The most effective interventions involved combining immediate pre-departure testing (0 h) with 14-day quarantine, achieving a 98% reduction in missed cases with any departure country risk classifications or universal measures. To implement a missed cases reduction threshold target of at least 80%, combinations should include quarantine for at least five days plus a vaccination certificate, regardless of departure country risk classification and testing. The vaccination certificate requirement can be waived if quarantine is increased to at least seven days, regardless of departure country risk classification and testing.

### Sensitivity analysis and model validation

Cluster analysis validation demonstrated moderate to good clustering quality for the three-cluster solution (Table S10). The average silhouette width of 0.439 indicated reasonable cluster separation for meaningful clustering structure [28]. The Calinski-Harabasz index of 91.62 supported the validity of the identified cluster boundaries. Although the Dunn index of 0.052 indicated relatively proximal clusters, this was mitigated by high algorithmic consensus, with ARIs of 0.782 and 0.829 for K-means versus Wards’s hierarchical clustering method and PAM, respectively, demonstrating robust cluster identification across multiple clustering algorithms and that the three clusters were generalizable and represented the true structure of the datasets.

The sensitivity analysis of the proportion of initial numbers of exposed to infectious compartments demonstrated that relative to baseline conditions (50% to 50%), both minimum and maximum interventions remained robust when varying the proportion to infectious 25% to 75% and 75% to 25%, showing negligible impact on missed cases and supporting the validity of the baseline simulation (Table S11). Similarly, the inclusion of 5% false-positive results in the susceptible and recovered compartments had minimal effects on either intervention strategy, confirming that disregarding false-positives in the model was acceptable (Table S12). Simulation based on 2019 historical cluster-specific actual numbers of travelers [29] as an initial population were convertible to the same rate as baseline (Table S13).

The model’s performance in predicting weekly imported cases per 100,000 travelers varied across PHPIT implementation periods (Figures S7 and S8). Reasonable agreement with reported data was observed during Periods B, C, and D, evidenced by most datapoints residing within the predicted range and clustering near the model mean. In Period A (May 2020–March 2021), the model exhibited a consistent overestimation, with reported cases consistently at or below the lower bounds. Period D (late 2021) showed larger deviations, suggesting reduced model reliability as policies evolved.

## Discussion

We aimed to determine the impacts of PHPIT, with a specific focus on its implementation, based on clustering travelers’ departure countries by risk levels. Cluster analysis identified Cluster 3, predominantly comprising low- and middle-income countries from Asia and Africa, as having the lowest infection rates but paradoxically the lowest vaccination coverage among the clusters. A previous study also reported no clear association between vaccination coverage and incidence rates [30]. Furthermore, Cluster 3 showed the greatest reduction in missed cases based on risk within the same scenarios, at 35%. During the four periods of border reopening, travelers from selected countries were permitted to enter, including the United Arab Emirates and Israel (from Cluster 1) and Singapore (from Cluster 2); these countries were in clusters with higher infection rates and fewer missed cases among departure countries based on risk. Comparing actual policy implementation with our model’s findings suggested that departure country risk classification might have prioritized criteria beyond infection rates and vaccination coverage in a departure country. The cluster analysis utilized seven-day averaged infection rates to prioritize timeliness in assessing active transmission intensity for real-time risk stratification. However, the use of cumulative data may provide longer-term epidemiological trends of the pandemic [31].

Classification may be improved if additional factors, such as testing capacities, reporting practices, and vaccine coverage levels, were considered in departure country risk classification and infection rate assessments. These factors can highlight infrastructure disparities among countries related to the COVID-19 pandemic, which could impact the risk of transmission and infection [32]. Furthermore, the input characteristic variables used in departure country risk classification may be influenced by factors beyond public health [33]. Infection rates were found to fluctuate due to the interventions and behaviors of each country’s population. Vaccine-induced protection was intermittent and intertwined with natural immunity gained from prior infections [34]. Therefore, policies targeting specific countries should be based on diverse public health data to accurately reflect the performance of control measures, prevent case importation, and avoid unnecessary discrimination. Although in this study we simplified the classification of travelers, it emphasizes the importance of recognizing heterogeneity among travelers, including travelers’ characteristics, travel type, destinations, distance, exposure risks, and transportation choices [35]. Further research is essential to assess how these criteria impact case importation, public health, and the economy. Exploring diverse variables, such as demographics, political systems, and economics, during the clustering process could provide valuable insights into PHPIT outcomes.

In our model, the impacts of requiring a vaccination certificate were influenced by vaccine efficacy and coverage in departure countries. Requiring vaccination certificates assumes all travelers are vaccinated, whereas when a vaccination certificate is not required, this will include a mix of vaccinated and unvaccinated individuals. The primary benefits of vaccination include preventing infection, reducing transmission, and providing protection against symptomatic illness, severe disease, and death [36, 37]. According to our model, severely symptomatic individuals, who are estimated to comprise 18% of infections, would typically be considered unable to travel and were thus excluded from the model. Among infected travelers, vaccinated individuals are likely to continue traveling if symptoms are mild or insufficiently disruptive. Vaccinated individuals exhibit reduced transmissibility, particularly when interacting with other vaccinated individuals [38]. This underscores the critical role of vaccination in minimizing transmission among travelers. This dynamic led to fewer travelers being excluded under the scenarios when a vaccination certificate was required than when one was not required. Despite these benefits, our findings suggest that requiring a vaccination certificate alone may not be a sufficiently robust measure to significantly reduce missed cases. Additional strategies are necessary to complement vaccination requirements and enhance their effectiveness in controlling case importation.

According to our model, testing reduced missed cases by just 4–5% when performed 72 h pre-departure. This limited reduction may be attributed to the incubation period of COVID-19. Assuming exposure occurred 1–3 days prior to testing, many exposed travelers are likely still within the incubation period on the day of testing [39], so some infected individuals will remain undetected and continue traveling. Immediate pre-departure testing was more effective than the 72, 48, and 24 h pre-departure testing options, reducing missed cases by 13–15%. Despite its efficacy, immediate pre-departure testing faces operational constraints due to the 24–48-hour processing time required for COVID-19 PCR tests [40]. Conversely, a single measure, entry testing (i.e., the test-and-go policy) could achieve a 29% reduction in missed cases. Thailand implemented this policy in March 2021, stipulating that travelers must have received a negative result 72 h pre-departure and have been vaccinated. Our model suggested this policy could reduce missed cases by 45%. However, the pre-departure testing requirement was revoked in April 2021 to facilitate tourism [41]. Combining testing on entry with a vaccination certificate requirement and departure country risk classification could achieve a maximum reduction of 50%, provided there was adequate healthcare capacity to manage potential transmission risks from missed cases and sufficient capacity for testing on arrival [42]. This would balance the need to control the spread of infection with the economic advantages of reduced disruption to travel and trade.

Our model suggests quarantine alone could effectively reduce missed cases by 45% with just a one-day quarantine period. Extending this to five days further reduced missed cases by at least 80%, regardless of vaccination requirement. This was because quarantine allows exposed individuals to become infectious, reaching a detectable viral load [43, 44] and enabling their identification through testing and preventing further transmission. However, longer quarantine periods may have a negative impact, particularly when the marginal cost extends beyond the quarantined individuals and affects the economy more broadly [45]. Moreover, the absence of government subsidies placed the entire financial burden of quarantine on travelers [27, 46]. When combined with hotels recouping enhanced operational expenditures (hygiene/staffing) via quarantine fees, this constituted a demonstrable economic disincentive for prospective visitors to Thailand. Hence, the duration of quarantine should be carefully adjusted, based on real-time monitoring of the domestic transmission situation and by implementing other PHPIT measures, such as a vaccination certificate requirement, to balance economic and public health considerations. Additionally, to exempt international travelers from quarantine measures, restrictive internal public health measures could mitigate the risk associated with open borders when the exemptions are applied to travelers from countries with similar epidemiological conditions [47].

Our findings highlight the unfavorable nature of departure country classification, as it showed no significant improvement in missed case detection compared with universal measures (Fig. 3). Restricting entry to travelers from countries belonging to any cluster yields the greatest reduction in missed cases, as lower infection rates within clusters do not necessarily translate to fewer missed cases. This outcome is influenced by various factors and characteristics specific to each departure country and cluster of departure countries. Consequently, only targeting countries with low infection rates may not always result in the lowest numbers of missed cases. Intervention combinations may vary; however, the differences among clusters of departure countries grouped by risk remain minimal. Therefore, selecting specific countries from which travelers are allowed to enter yields no significant advantage over a universally applied approach. Infections that occurred during travel emerged as a critical factor contributing to increased case numbers, particularly among unvaccinated individuals. This highlights the importance of considering vaccination status and in-transit transmission risks when designing policies that can effectively reduce missed cases. However, when requiring a vaccination certificate was combined with other interventions, the additional reduction was minimal, particularly when further quarantine days were added. Therefore, if quarantine is progressively implemented, vaccination certificate requirement and departure country risk classification can be omitted without compromising a reduction in missed cases.

From the perspective of health equity and ethical justification, international regulations, cooperation, and recommendations should be considered. Travel bans or restrictions targeting affected areas are often ineffective in preventing case importation and can adversely impact socioeconomic development and perpetuate stigma and discrimination [48], while tightening border controls and restricting travel can disproportionately affect vulnerable populations, exacerbating discrimination and xenophobia [49]. Imposing blanket measures targeting particular nationalities or applying vaccination status requirements without clear evidence of health risk would constitute discriminatory practices [50, 51].

Based on our findings, quarantine can achieve considerable reductions in missed cases while avoiding unnecessary and potentially discriminatory policies. However, quarantine remains one of the least favorable policies for cross-border travel, as it negatively impacts tourism and travel demand, with substantial economic consequences [52]. Adjusting the duration of quarantine and requiring a vaccination certificate may mitigate this. Bespoke combinations of PHPIT could be implemented, provided that pre-entry processing and screening exist to reduce administrative workloads. Identifying optimal policies for border reopening or cross-border travel interventions during any future pandemic, particularly for respiratory diseases, will require careful balancing of public health priorities, economic considerations, and ethical perspectives.

Limitations of our study included the lack of transmission data throughout the period of traveling which prevented parameter estimation via model fitting. Consequently, while model-estimated imported case counts were validated against reported data and found to be consistent, direct validation of missed case estimates was impossible. Furthermore, the model disregarded ground-based travelers, who constitute a substantial proportion of cross-border movement, particularly from neighboring countries. The cluster analysis relied on just three variables (infection rate, testing positivity, and vaccination coverage) from a limited seven-day retrospective period, from 111 out of 260 countries, i.e., more than half of all countries were excluded due to incomplete data, limiting the model’s ability to fully represent global patterns. Lastly, we used a deterministic model structure, which was unable to capture some of the randomness of individual infections and transmission processes.

## Conclusion

In the Thai context, exclusively granting entry permission to travelers from specific departure countries or to vaccinated individuals showed no significant difference in reducing missed cases compared with policies that were applied universally. Given Thailand’s robust domestic healthcare infrastructure, quarantine prior to community integration (with exit testing) may represent an effective standard intervention that could be implemented in combination with other interventions to optimize PHPIT policy outcomes and minimize missed cases. While this would help safeguard public health and preserve economic security, it would necessitate a thorough pre-implementation evaluation to address and mitigate potential adverse effects across all dimensions and ensure that any policies were effective and equitable. Future PHPIT implementation should include pre-evaluation to ensure interventions are effective while avoiding unnecessary discrimination against international travelers, to promote equity while safeguarding public health and economic security.

## Supplementary Information

Supplementary Material 1.

## Data Availability

We used data on total monthly travelers provided by the Bank of Thailand [9], along with data on reported cases of COVID-19 from the Department of Disease Control [53] and COVID-19 datasets from Our World in Data [18]. The original datasets are available on the owners’ websites. The processed datasets used in analysis, data generated from the model simulations, datasets supporting the conclusions of this article, as well as the model calculator, which was a tool created to simulate the model, are all available in the CBC model repository: https://github.com/mvidhyagorn/CBC_Model.

## Abbreviations

CBC: Cross-Border Control
COE: Certificate of Entry
COVID-19: Coronavirus disease 2019
GDP: Gross Domestic Product
PHPIT: Public Health Policy for International Travelers
SEIR: Susceptible, Exposed, Infectious, Recovered
