# Supplementary material for: Mathematical modeling of public health policy for international travelers (PHPIT): a case study of the COVID-19 pandemic in Thailand
Source: BMC Public Health. 2025 Dec 1;26:68. doi: 10.1186/s12889-025-25811-5 (PMC12777338; doi:10.1186/s12889-025-25811-5)
Supplement: Supplementary file 1 — Supplementary Material 1. [file 12889_2025_25811_MOESM1_ESM.docx]

# **Mathematical modeling of public health policy for international travelers (PHPIT): A case study of the COVID-19 pandemic in Thailand**

**Supplementary materials**

| **Name** | **Descriptions** |
| --- | --- |
| Table S1 | Operations and case definitions |
| Figure S1 | COVID-19 precautionary measures and requirements for traveling to Thailand |
| Table S2 | The four periods of Thailand’s border reopening, with key interventions for the periods, including entry policy descriptions, allowed countries, pre-departure testing, entry testing, vaccine status, and quarantine requirements |
| Table S3 | Numbers of imported cases of COVID-19 (monthly) from January 2020 to August 2022, numbers of travelers (monthly) from January 2019 to August 2022, and numbers of imported cases of COVID-19 per 100,000 travelers |
| Table S4 | Parameters, symbols, and values for the model simulation |
| Table S5 | Difference equations for the intervention sub-model |
| Figure S2 | Calculation sheet used to solve difference equations, showing parameters, discretized equations, and notation and relationships between columns, rows, and cells |
| Figure S3 | Screenshot of the dashboard sheet of the simulation on an Excel spreadsheet, including parameter values, scenario selection, and a results table providing predicted numbers of imported cases before entry and after being quarantined |
| Figure S4 | Contributions to principal components PC1 and PC2 |
| Figure S5 | Comparison of determined optimal numbers of clusters between the elbow method, the gap statistic method, and the silhouette method (K = 3) |
| Figure S6 | Risk classification of departure countries using K-means clustering analysis based on the country-specific infection rate, testing positivity, and vaccine coverage, between 1 January 2020 and 9 January 2022 |
| Table S6 | Countries comprising the various clusters of departure countries, categorized by risk |
| Table S7 | Model-estimated numbers of missed cases (leaked to communities) per 100,000 travelers per week in 336 scenarios of a combination of public health policies for international travelers |
| Table S8 | Model-estimated numbers of COVID-19 infected travelers detected after arrival (imported cases) per 100,000 travelers per week in 336 scenarios of a combination of public health policies for international travelers |
| Table S9 | Percentage reduction in missed cases (leaked to communities) per 100,000 travelers per week in 336 scenarios of a combination of public health policies for international travelers |
| Table S10 | Cluster analysis validation metrics and results |
| Table S11 | Sensitivity analysis of initial numbers of exposed and infectious compartments |
| Table S12 | Sensitivity analysis of false-positive results in susceptible and recovered compartments |
| Table S13 | Comparison of missed cases estimation: Cluster-specific traveler numbers vs fixed numbers assumption |
| Table S14 | List of risks identified for imported cases reported between April 2020 and April 2022 |
| Table S15-A, B | List of key PHPITs during the four periods with estimated imported cases for model validation |
| Figure S7 | Rate of missed cases per 100,000 travelers per week by case type compared with model data for the same rate within a similar period of combinations of PHPIT options |
| Figure S8 | Rate of missed cases per 100,000 travelers per week by cluster type compared with model data for the same rate within a similar period of combinations of PHPIT options |

**Table S1** Operations and case definitions

| Imported case | The World Health Organization (WHO) defined patients with confirmed COVID-19 who acquired SARS-CoV-2 infection outside of the location where they were diagnosed as an imported case [1]. This definition was primarily epidemiological and differentiated from local or domestic cases. The term “location” or “area” may refer to a specific region within a country, as seen in studies that define a case imported from another province within the country as an imported case [2]. However, in this study, the term imported case refers to a COVID-19 infection that was acquired outside of Thailand before the individual arrived, regardless of the individual’s nationality, and was reported as a COVID-19 case in Thailand. |
| --- | --- |
| Missed case | A missed case was a traveler who was exposed to or infected with COVID-19 but was not identified or whose infection was not detectable due to minimal symptoms, asymptomatic presentation, or undetectable viral load during the time they were subject to measures arising from the public health policy for international travelers (PHPIT). The number of missed cases was calculated using a set of equations and demonstrated an inverse relationship to the number of imported cases. Missed cases were quantified as the number of missed cases per 100,000 travelers per week. In this study, we presented the percentage reductions in missed cases when considering various scenarios compared with taking no action. |
| Certificate of entry | In Thailand’s case, this was later referred to as the “Thailand Pass”. This was a document that served as proof that an individual had completed the cross-border travel control requirements and was allowed to enter Thailand. All travelers arriving in Thailand by air and ground were required to register with a Thai consulate or embassy in their home country and obtain a Thailand Pass before traveling to Thailand. The documents required for registration included a vaccination certificate, a passport, and medical insurance [3]. |
| Quarantine | A public health measure used to prevent the spread of communicable diseases by separating people who have been in close contact with an infected person from the general population; quarantine may take place in their own home or at a place arranged by a health authority. It was one of the initial measures implemented during the COVID-19 pandemic until vaccines became available. Quarantine usually takes place in the home and may be applied at the individual level or to a group or community of individuals who have been exposed [4, 5]. |
| Pre-departure testing | A cross-border travel control requirement adopted by governments worldwide, typically involving RT-PCR testing to verify that individual travelers are free of COVID-19. Pre-departure testing was not a required document for a certificate of entry (Thailand Pass), but travelers were required to present proof of testing at their departure airport and at Thailand’s border control before being allowed to enter the country. Normally, testing had to be performed within 72 hours prior to departure, but in this study, we varied the testing time to 48 or 24 hours prior to departure, or even immediately before departure. |
| Vaccination certificate | A document that records a vaccine received by an individual. It typically includes information such as the individual’s name, the vaccine administered, the date it was administered, and any other relevant data. During the COVID-19 pandemic, WHO proposed the use of electronic COVID-19 vaccination certificates as a way to digitally document an individual’s COVID-19 vaccination status [6]. A vaccination certificate was a document that was required to register for the Thailand Pass, which was a requirement for entering Thailand and was considered a form of cross-border travel control. However, this requirement was cancelled on 1 October 2022 [3]. |
| Departure country | A country or territory where foreign travelers or Thai citizens must first register for a certificate of entry before traveling to Thailand. This could be either the country of origin where the foreign traveler or Thai citizen resides, or the country they last visited before coming to Thailand. |
| Air travel | A form of travel, in an aircraft and via airports, for the purpose of flying as a passenger. Despite being densely populated spaces, air travel on aircraft was claimed to have a very low risk of transmission, and steps were taken to mitigate risks while flying, such as an onboard ventilation system that circulated and refreshed the air supply, wearing of facemasks by passengers and crew, distancing during boarding, disinfection of high-touch aircraft surfaces, and passengers’ attestations of not having COVID-19-related symptoms and commitment to adhere to the airline’s mask policy [7, 8]. |

**Figure S1** COVID-19 precautionary measures and requirements for traveling to Thailand [9] (SQ = state quarantine; ASQ = alternative state quarantine; AHQ = alternative hospital quarantine).


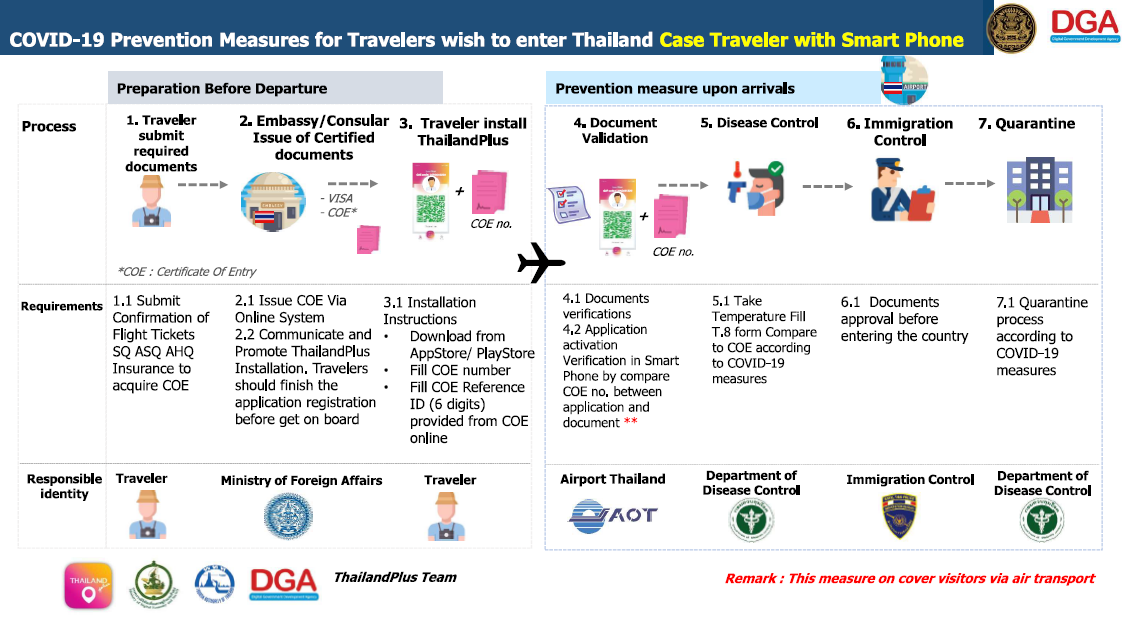


**Table S2** The four periods of Thailand’s border reopening with key interventions for the periods including entry policy descriptions, allowed countries, pre-departure testing, entry testing, vaccine status, and quarantine requirements [10–16] (ASQ = alternative state quarantine; AHQ = alternative hospital quarantine; COE = certificate of entry; RT-PCR = reverse transcriptase polymerase chain reaction, UK = United Kingdom; USA = United States of America)

|  |  |  |  |  |  |  |  |
| --- | --- | --- | --- | --- | --- | --- | --- |
| **Period** | **Period** | **Entry Policy Description** | **Allowed Countries / Groups** | **Pre-departure Testing** | **Entry Testing** | **Vaccine Status** | **Quarantine Requirement** |
| **Period A** | **Jan-Mar 2021** | Complete closure except for Thais and essential exceptions; full ASQ/AQ quarantine | Thai nationals, residents, essential/emergency cases, diplomats, work permit holders, family of Thais | RT-PCR 72h pre-departure | RT-PCR on arrival | Not required; vaccination not yet a criterion | 14 days in ASQ/AQ facility |
| **Period B** | **Apr-Jun 2021** | Gradual reduction of quarantine for vaccinated (April), reversal to 14 days (May/June), COE required | COE holders: diplomats, essential personnel, expats, family of Thais (no general country list for tourism) | RT-PCR 72h pre-departure | RT-PCR on arrival | April: vaccination reduced quarantine (7 days); afterwards, recorded but did not exempt | April: 7-10 days (vaccinated); May/June: 14 days |
| **Period C** | **Jul-Oct 2021** | Sandbox: quarantine-free for fully vaccinated from approved list; AQ remains for others | Fully vaccinated from 69 approved countries for Sandbox (e.g. Australia, Canada, China, France, Germany, Japan, UK, USA, etc.); AQ for other categories | RT-PCR 72h pre-departure | RT-PCR on arrival, during stay | Full vaccination required (WHO/Thai FDA approved) | Sandbox: Must stay in area 14 days, no quarantine; AQ: 7-14 days others |
| **Period D** | **Nov 2021-Jun 2022** | "Test & Go": Quarantine-free for vaccinated from 63 countries (Nov), expanding to ALL countries (Feb); AQ for others | Nov 2021: Fully vaccinated from 63 approved countries (similar to Sandbox); Feb 2022 onward: all countries permitted for fully vaccinated travelers; AQ for unvaccinated | RT-PCR 72h pre-departure | RT-PCR on arrival; additional testing as required | Full vaccination required for Test & Go/Sandbox | 1 night (pending test result); AQ 7-10 for others |
|  |  |  |  |  |  |  |  |

**Table S3** Numbers of imported cases of COVID-19 (monthly), numbers of travelers (monthly) from April 2020 to April 2022, and numbers of imported cases of COVID-19 per 100,000 travelers.

**Table S4** Parameters, symbols, and values for the model simulations

| **Parameter** | **Symbol** | **Value** | **Source/reference** |
| --- | --- | --- | --- |
| Applicants for certificate of entry (COE) | $N$ | 100,000 | Assumed |
| Cluster infection rate  (per million per day) | $\alpha$ | Universal = 196.70  C1 = 142.09  C2 = 650.58  C3 = 48.72 | Calculated  (see Table 1) |
| Infection rate during travel | $\partial$ | $\alpha*2$ | Assumed |
| False-negative testing during the latency period | $\left( 1- \mu\right)$ | 0.84 | [17] |
| False-negative testing during the infectious period | $\left( 1-\tau\right)$ | 0.29 | [17] |
| Vaccine efficacy against transmission | ϑ | 0.3 | [18]  Based on WHO’s recommended minimum vaccine efficacy at 50%, projected to reduce infection by 23-46%. |
| Vaccine-associated reductions in transmission in population level  When vaccination certificate is required  $\sigma=\omega\times\left( 1-\vartheta\right)$  When vaccination certificate is NOT required  $\sigma=\omega\times\left( 1-\vartheta\right)+\left( 1-\omega\right)$ | $\sigma$ | 0.7 – 0.97 | Calculated |
| Vaccine efficacy against severe disease | δ | 0.7 | [19] |
| Vaccine-associated reductions in severity in population level  When vaccination certificate is required $\rho=\omega\times\left( 1-\delta\right)$  When vaccination certificate is NOT required  $\rho=\omega\times\left( 1-\delta\right)+\left( 1-\omega\right)$ | $\left( \rho\right)$ | 0.30 – 0.93 | Calculated |
| Rate of being infectious  (fraction of incubation period) or  $\frac{1}{incubation period}$ ;  Incubation period is approximately 4 days | $\beta$ | 0.25 | [20]  95% CI = 1/3.5 – 1/4.5 |
| Rate of recovery  (fraction of infectious period) or  $\frac{1}{infectious period}$ ;  The infectious period is 14 days | $\gamma$ | 0.0714 | [21]  95^th^ percentile = 14 days |
| Probability of severe symptomatic infection | $\pi$ | 0.18 | [22]  (moderate to severe symptoms 18-20%) |
| Vaccination coverage (%) | $\omega$ | Universal = 42.83  C1 = 65.81  C2 = 40.90  C3 = 19.15 | Calculated  (see Table 1) |

**Table S5** Difference equations for the intervention sub-model

| **Stage** | **Equations** |
| --- | --- |
| **Initial** | $S_{initial}=100000-E_{initial}-I_{initial}-R_{initial}$  $E_{initial}=\alpha\left( \frac{N}{2} \right)$  $I_{initial}=\alpha\left( \frac{N}{2} \right)$  $R_{initial}=0$ |
| **P1** | $S_{p1(t)}=S_{initial}-\alpha\sigma S_{p1(t-1)}$  $E_{p1(t)}=E_{initial}-\beta E_{p1(t-1)}$  $I_{p1(t)}=I_{initial}-\gamma I_{p1(t-1)}-\pi I_{p1(t-1)}\left( 1-\rho\right)$  $R_{p1(t)}={R_{initial}-R}_{p1(t-1)}$ |
| **P2** | $S_{p2\left( t \right)}=S_{p1\left( t-1 \right)}-\alpha\sigma S_{p1(t-1)}$  $E_{p2(t)}=E_{p1(t-1)}+\alpha\sigma S_{p1(t-1)}-\beta E_{p1(t-1)}-E_{p1(t-1)}\left( 1-\mu\right)$  $I_{p2(t)}= \beta E_{p1(t-1)}+I_{p1(t-1)}-\pi I_{p1(t-1)}\left( 1-\rho\right)-I_{p1(t-1)}\left( 1-\tau\right)-\gamma I_{p1(t-1)}$  $R_{p2(t)}=R_{p1}+\gamma I_{p1}$ |
| **P3** | $S_{p3(t)}=S_{p2(t-1)}-\alpha\sigma S_{p2(t-1)}$  $E_{p3(t)}=E_{p2(t-1)}+\alpha\sigma S_{p2(t-1)}-\beta E_{p2(t-1)}$  $I_{p3(t)}= \beta E_{p2(t-1)}+I_{p2(t-1)}-\pi I_{p2(t-1)}\left( 1-\rho\right)-{\gamma I}_{p(t-1)}$  $R_{p3(t)}=R_{p2(t-1)}+\gamma I_{p2(t-1)}$ |
| **P4** | $S_{p4(t)}=S_{p3(t-1)}-\partial\sigma S_{p3(t-1)}$  $E_{p4(t)}=E_{p3(t-1)}+\alpha\sigma S_{p3(t-1)}-\beta E_{p3(t-1)}$  $I_{p4(t)}= \beta E_{p3(t-1)}+I_{p3(t-1)}-\pi I_{p3(t-1)}\left( 1-\rho\right)-{\gamma I}_{p3(t-1)}$  $R_{p4(t)}=R_{p3(t-1)}+\gamma I_{p3(t-1)}$ |
| **A** | $S_{A(t)}=S_{p4(t-1)}$  $E_{A(t)}=E_{p4(t-1)}+\partial\sigma S_{p4(t-1)}-E_{p4(t-1)}\left( 1-\mu\right)-\beta E_{p4(t-1)}$  $I_{A(t)}= \beta E_{p4(t-1)}+I_{p4(t-1)}-\pi I_{p4(t-1)}\left( 1-\rho\right)-{\gamma I}_{p4(t-1)}$  $R_{A(t)}=R_{p4(t-1)}+\gamma I_{p4(t-1)}$ |
| **Q** | $S_{Q(t)}=S_{A(t-1)}$  $E_{Q(t)}=E_{A(t-1)}-E_{A(t-1)}\left( 1-\mu\right)$  $I_{Q(t)}= \beta E_{A(t-1)}+I_{A(t-1)}-\pi I_{A(t-1)}\left( 1-\rho\right)-I_{A(t-1)}\left( 1-\tau\right)$  $R_{Q(t)}=R_{A(t-1)}+\gamma I_{A(t-1)}$ |
| **Missed cases** | ${MC}_{(t)}=E_{p(t)}+I_{p(t)}$ |

**Figure S2** Calculation sheet used to solve the difference equations, showing parameters, discretized equations, and notation and relationships between columns, rows, and cells

**
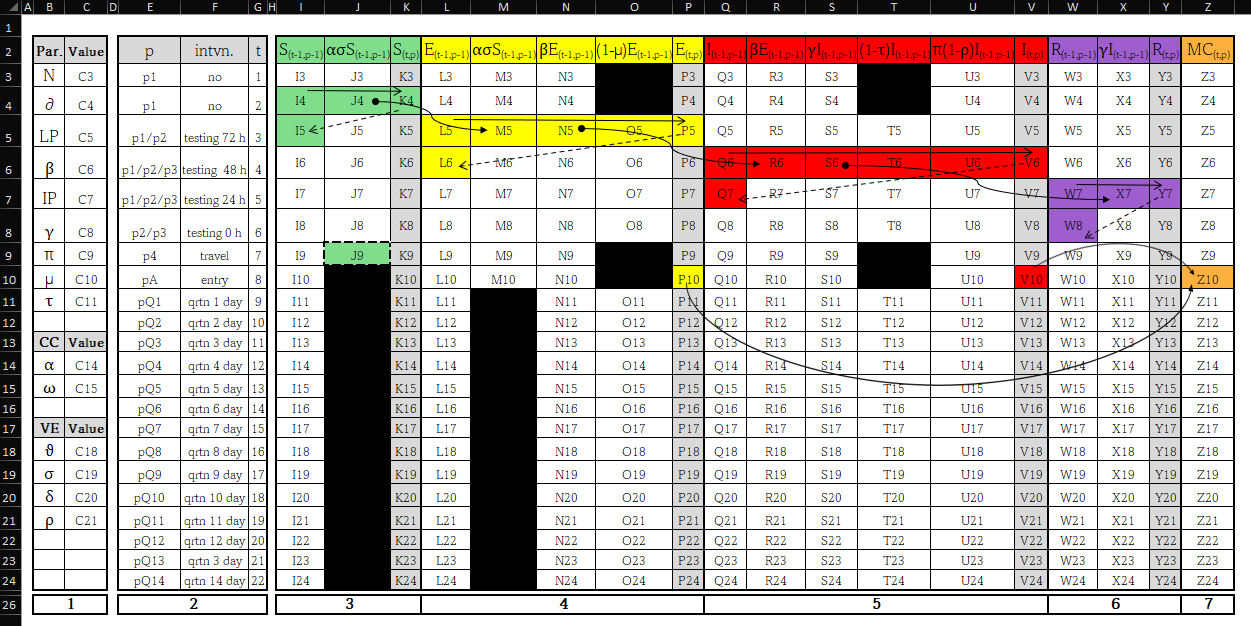
**

**Figure S3** Screenshot of the dashboard sheet of the simulation on an Excel spreadsheet, including parameter values, scenario selection, and a results table providing predicted numbers of imported cases before entry and after being quarantined. (The estimated number of infected travelers entering communities (missed case) was derived by simulating the combined impacts of before and after arrival to Thailand, including departure-country risk classification, vaccination certificate requirements, pre-departure testing, entry testing, and quarantine. Estimated numbers of missed case was calculated for both the “Before entry” and “After entry”, with final estimated missed case entering community corresponding to the “After entry” period under selected quarantine options and cluster of departure country based on risk.)


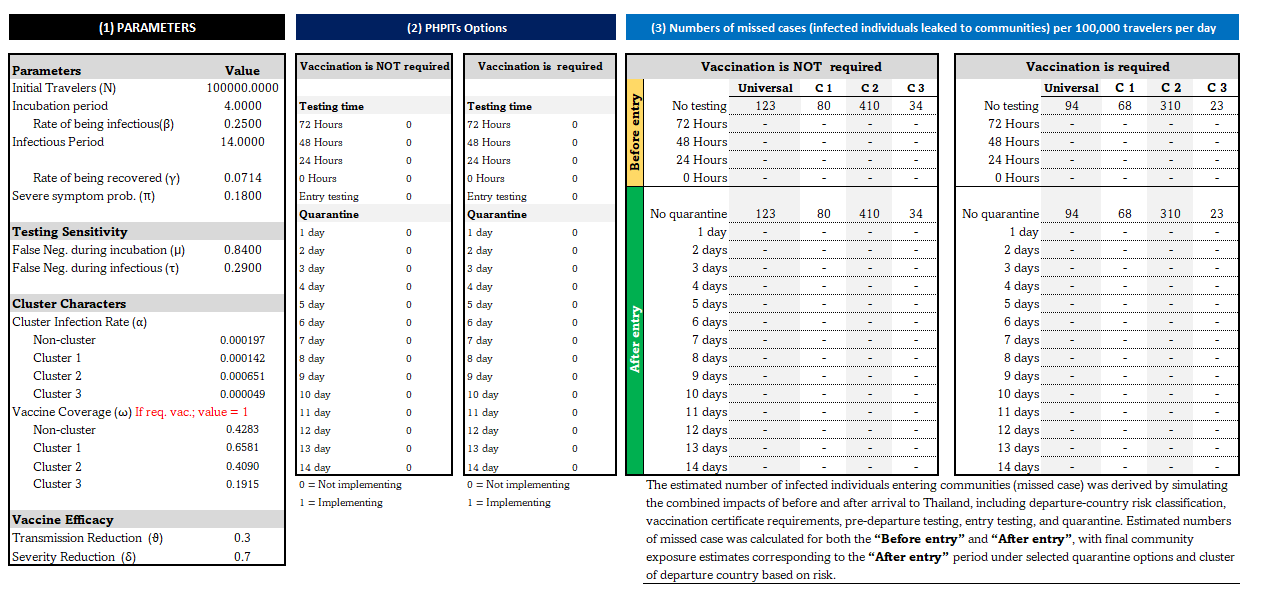


**Figure S4** Contributions to principal components PC1 and PC2 (vac = vaccination coverage; pos = positivity testing probability; infec = infection rate)

**
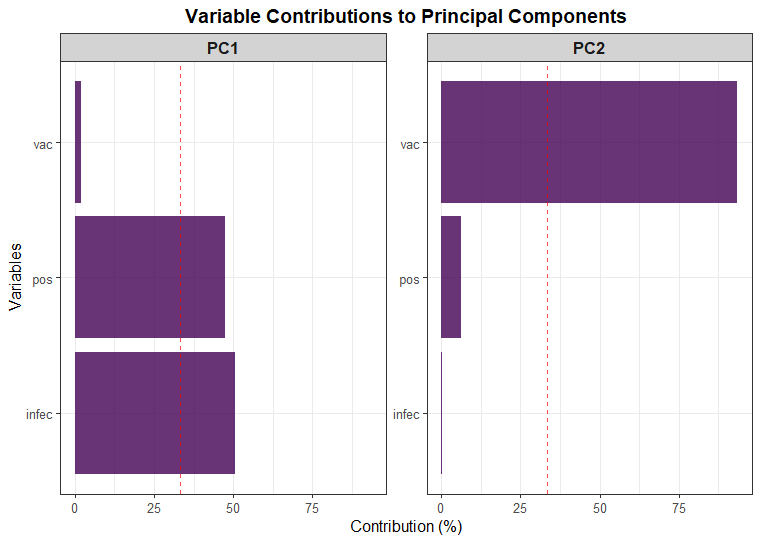
**

**Figure S5** Comparison of determined optimal numbers of clusters between the Elbow method, the Gap statistic method, and the Silhouette method (K = 3)

**
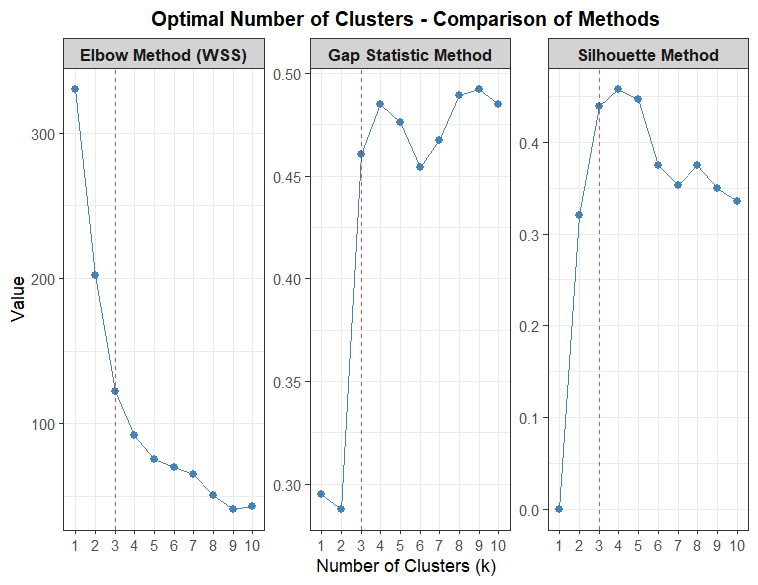
**

**Figure S6** Risk classification of departure countries using K-means cluster analysis based on the country-specific infection rate, testing positivity, and vaccine coverage, between 1 January 2020 and 9 January 2022. (ISO Country Code [23]; ARE = United Arab Emirates; BIH = Bosnia and Herzegovina; BLR = Belarus; CAN = Canada; CIV = Cote d'Ivoire; CYP = Cyprus; CZE = Czechia; DOM = Dominican Republic; GBR = United Kingdom; GNQ = Equatorial Guinea; IRQ = Iraq; KNA = Saint Kitts and Nevis; KOR = South Korea; LUX = Luxembourg; MMR = Myanmar; NAM = Namibia; NGA = Nigeria; NPL = Nepal; NZL = New Zealand; PAK = Pakistan; PAN = Panama; PHL = Philippines; SAU = Saudi Arabia; SEN = Senegal; SWE = Sweden; UGA = Uganda; USA = United States; VCT = Saint Vincent and the Grenadines; ZAF = South Africa; ZWE = Zimbabwe; Cluster 1 = 47 countries, Cluster 2 = 20 countries, Cluster 3 = 44 countries)


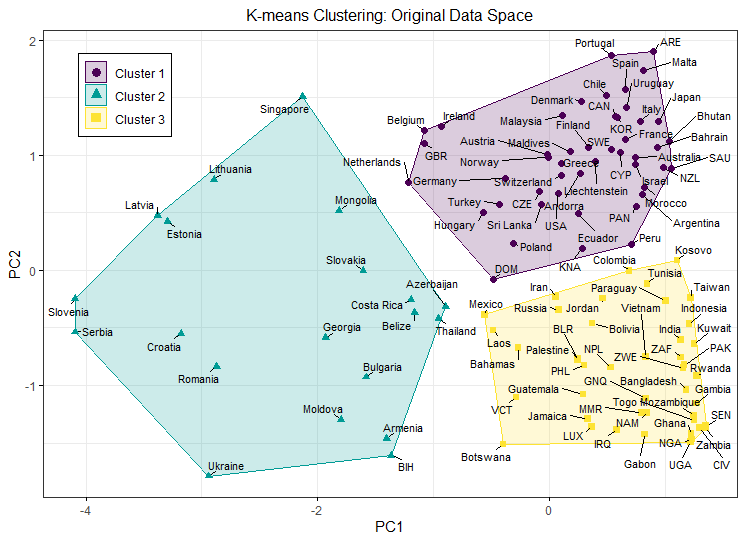


**Table S6** Countries comprising the various clusters of departure countries categorized by risk.

| **Cluster 1 (47 countries)** |
| --- |
| Andorra, Argentina, Australia,, Bahrain , Belgium, Bhutan , Canada , Chile , Cyprus , Czechia , Denmark, Dominican Republic , Ecuador , Finland , France , Germany, Greece , Hungary , Ireland , Israel , Italy , Japan, Liechtenstein , Malaysia , Maldives , Malta , Morocco, Netherlands , New Zealand , Norway , Panama , Peru, Poland , Portugal , Saint Kitts and Nevis , Saudi Arabia, South Korea , Spain , Sri Lanka , Sweden , Switzerland, Turkey , United Arab Emirates , United Kingdom, United States , Uruguay |
| **Cluster 2 (20 countries)** |
| Armenia, Azerbaijan, Belize, Bosnia and Herzegovina, Bulgaria, Costa Rica, Croatia, Estonia, Georgia, Latvia, Lithuania, Moldova, Mongolia, Romania, Serbia, Singapore, Slovakia, Slovenia, Thailand, Ukraine |
| **Cluster 3 (44 countries)cal** |
| Bahamas , Bangladesh , Belarus , Bolivia , Botswana, Colombia , Cote d'Ivoire , Equatorial Guinea , Gabon , Gambia, Ghana , Guatemala , India , Indonesia , Iran , Iraq, Jamaica , Jordan , Kosovo , Kuwait , Laos , Luxembourg, Mexico , Mozambique , Myanmar , Namibia , Nepal, Nigeria , Pakistan , Palestine , Paraguay , Philippines, Russia, Rwanda , Saint Vincent and the Grenadines , Senegal, South Africa , Taiwan , Togo , Tunisia , Uganda, Vietnam , Zambia , Zimbabwe |

**Table S7** Model estimated numbers of missed cases (leaked to communities) per 100,000 travelers per week in 336 scenarios of combination of public health policy for international travelers.

| **Testing** | **Vaccination Certificate Requirements** | **Quarantine (Days)** | **Universal** | **Cluster  1** | **Cluster  2** | **Cluster  3** |
| --- | --- | --- | --- | --- | --- | --- |
| **No Testing** | **Not required** | **No** | *863* | *560* | *2,872* | *238* |
|  |  | **1** | *473* | *306* | *1,576* | *131* |
|  |  | **3** | *359* | *226* | *1,197* | *102* |
|  |  | **5** | *245* | *148* | *819* | *73* |
|  |  | **7** | *170* | *98* | *573* | *54* |
|  |  | **10** | *102* | *54* | *347* | *36* |
|  |  | **14** | *55* | *25* | *187* | *22* |
|  | **Required** | **No** | *657* | *474* | *2,167* | *163* |
|  |  | **1** | *358* | *258* | *1,182* | *89* |
|  |  | **3** | *256* | *184* | *843* | *64* |
|  |  | **5** | *158* | *114* | *521* | *39* |
|  |  | **7** | *98* | *71* | *323* | *24* |
|  |  | **10** | *48* | *35* | *159* | *12* |
|  |  | **14** | *19* | *14* | *62* | *5* |
| **72 hours pre-departure** | **Not required** | **No** | *822* | *536* | *2,734* | *226* |
|  |  | **1** | *462* | *300* | *1,538* | *128* |
|  |  | **3** | *351* | *222* | *1,170* | *100* |
|  |  | **5** | *239* | *145* | *799* | *71* |
|  |  | **7** | *166* | *96* | *558* | *52* |
|  |  | **10** | *100* | *53* | *338* | *35* |
|  |  | **14** | *53* | *25* | *182* | *21* |
|  | **Required** | **No** | *633* | *456* | *2,088* | *157* |
|  |  | **1** | *352* | *254* | *1,162* | *88* |
|  |  | **3** | *252* | *181* | *830* | *63* |
|  |  | **5** | *155* | *112* | *513* | *39* |
|  |  | **7** | *96* | *70* | *318* | *24* |
|  |  | **10** | *47* | *34* | *156* | *12* |
|  |  | **14** | *19* | *13* | *61* | *5* |
| **48 hours pre-departure** | **Not required** | **No** | *806* | *526* | *2,680* | *221* |
|  |  | **1** | *455* | *296* | *1,513* | *126* |
|  |  | **3** | *346* | *219* | *1,153* | *98* |
|  |  | **5** | *235* | *143* | *788* | *70* |
|  |  | **7** | *164* | *95* | *550* | *51* |
|  |  | **10** | *98* | *52* | *333* | *34* |
|  |  | **14** | *52* | *24* | *179* | *21* |
|  | **Required** | **No** | *621* | *447* | *2,048* | *154* |
|  |  | **1** | *347* | *250* | *1,145* | *86* |
|  |  | **3** | *249* | *179* | *820* | *62* |
|  |  | **5** | *153* | *111* | *506* | *38* |
|  |  | **7** | *95* | *69* | *314* | *24* |
|  |  | **10** | *47* | *34* | *154* | *12* |
|  |  | **14** | *18* | *13* | *61* | *5* |
| **24 hours pre-departure** | **Not required** | **No** | *779* | *509* | *2,590* | *213* |
|  |  | **1** | *444* | *289* | *1,478* | *122* |
|  |  | **3** | *339* | *215* | *1,128* | *96* |
|  |  | **5** | *230* | *140* | *770* | *68* |
|  |  | **7** | *160* | *93* | *537* | *50* |
|  |  | **10** | *96* | *51* | *325* | *33* |
|  |  | **14** | *51* | *24* | *175* | *20* |
|  | **Required** | **No** | *602* | *434* | *1,985* | *150* |
|  |  | **1** | *340* | *245* | *1,121* | *85* |
|  |  | **3** | *244* | *176* | *805* | *61* |
|  |  | **5** | *151* | *109* | *497* | *38* |
|  |  | **7** | *93* | *67* | *308* | *23* |
|  |  | **10** | *46* | *33* | *151* | *11* |
|  |  | **14** | *18* | *13* | *59* | *4* |
| **0 hours pre-departure** | **Not required** | **No** | *742* | *485* | *2,465* | *203* |
|  |  | **1** | *430* | *280* | *1,430* | *118* |
|  |  | **3** | *329* | *209* | *1,095* | *93* |
|  |  | **5** | *223* | *136* | *747* | *66* |
|  |  | **7** | *155* | *90* | *520* | *48* |
|  |  | **10** | *93* | *49* | *314* | *32* |
|  |  | **14** | *49* | *23* | *169* | *19* |
|  | **Required** | **No** | *575* | *415* | *1,898* | *143* |
|  |  | **1** | *330* | *238* | *1,090* | *82* |
|  |  | **3** | *238* | *172* | *785* | *59* |
|  |  | **5** | *147* | *106* | *485* | *37* |
|  |  | **7** | *91* | *66* | *301* | *23* |
|  |  | **10** | *45* | *32* | *148* | *11* |
|  |  | **14** | *18* | *13* | *58* | *4* |
| **Entry testing** | **Not required** | **No** | *615* | *402* | *2,044* | *168* |
|  |  | **1** | *405* | *266* | *1,347* | *111* |
|  |  | **3** | *300* | *191* | *998* | *84* |
|  |  | **5** | *203* | *125* | *678* | *60* |
|  |  | **7** | *140* | *82* | *471* | *43* |
|  |  | **10** | *84* | *45* | *283* | *28* |
|  |  | **14** | *44* | *21* | *152* | *17* |
|  | **Required** | **No** | *476* | *343* | *1,572* | *119* |
|  |  | **1** | *316* | *228* | *1,042* | *79* |
|  |  | **3** | *220* | *158* | *725* | *55* |
|  |  | **5** | *136* | *98* | *447* | *34* |
|  |  | **7** | *84* | *61* | *277* | *21* |
|  |  | **10** | *41* | *30* | *136* | *10* |
|  |  | **14** | *16* | *12* | *53* | *4* |
|  |  |  |  |  |  |  |

**Table S8** Model estimated numbers of COVID-19 infected travelers detected after arrival (imported case) per 100,000 travelers per week in 336 scenarios of a combination of public health policies for international travelers

| **Testing** | **Vaccination Certificate Requirements** | **Quarantine (Days)** | **Universal** | **Cluster  1** | **Cluster  2** | **Cluster  3** |
| --- | --- | --- | --- | --- | --- | --- |
| **No Testing** | **Not required** | **No** | *14* | *13* | *45* | *2* |
|  |  | **1** | *364* | *241* | *1,211* | *98* |
|  |  | **3** | *528* | *354* | *1,752* | *140* |
|  |  | **5** | *671* | *453* | *2,227* | *177* |
|  |  | **7** | *767* | *518* | *2,542* | *201* |
|  |  | **10** | *856* | *576* | *2,837* | *225* |
|  |  | **14** | *920* | *615* | *3,052* | *243* |
|  | **Required** | **No** | *23* | *16* | *75* | *6* |
|  |  | **1** | *290* | *209* | *959* | *72* |
|  |  | **3** | *434* | *313* | *1,433* | *108* |
|  |  | **5** | *558* | *402* | *1,840* | *139* |
|  |  | **7** | *636* | *458* | *2,099* | *158* |
|  |  | **10** | *703* | *507* | *2,321* | *175* |
|  |  | **14** | *745* | *537* | *2,458* | *185* |
| **72 hours pre-departure** | **Not required** | **No** | *12* | *12* | *38* | *2* |
|  |  | **1** | *332* | *221* | *1,102* | *89* |
|  |  | **3** | *491* | *332* | *1,628* | *129* |
|  |  | **5** | *632* | *429* | *2,093* | *165* |
|  |  | **7** | *725* | *492* | *2,401* | *189* |
|  |  | **10** | *812* | *549* | *2,690* | *212* |
|  |  | **14** | *874* | *588* | *2,899* | *230* |
|  | **Required** | **No** | *20* | *14* | *66* | *5* |
|  |  | **1** | *269* | *194* | *888* | *67* |
|  |  | **3** | *410* | *295* | *1,352* | *102* |
|  |  | **5** | *531* | *383* | *1,753* | *132* |
|  |  | **7** | *608* | *438* | *2,007* | *151* |
|  |  | **10** | *675* | *486* | *2,226* | *168* |
|  |  | **14** | *715* | *516* | *2,360* | *178* |
| **48 hours pre-departure** | **Not required** | **No** | *12* | *11* | *37* | *1* |
|  |  | **1** | *322* | *215* | *1,069* | *86* |
|  |  | **3** | *478* | *323* | *1,586* | *126* |
|  |  | **5** | *617* | *419* | *2,045* | *161* |
|  |  | **7** | *709* | *481* | *2,349* | *185* |
|  |  | **10** | *794* | *537* | *2,633* | *207* |
|  |  | **14** | *856* | *576* | *2,839* | *225* |
|  | **Required** | **No** | *19* | *14* | *64* | *5* |
|  |  | **1** | *261* | *188* | *862* | *65* |
|  |  | **3** | *399* | *288* | *1,318* | *99* |
|  |  | **5** | *519* | *374* | *1,713* | *129* |
|  |  | **7** | *595* | *429* | *1,964* | *148* |
|  |  | **10** | *661* | *476* | *2,180* | *164* |
|  |  | **14** | *701* | *505* | *2,313* | *174* |
| **24 hours pre-departure** | **Not required** | **No** | *11* | *10* | *34* | *1* |
|  |  | **1** | *304* | *203* | *1,010* | *81* |
|  |  | **3** | *456* | *309* | *1,512* | *120* |
|  |  | **5** | *592* | *402* | *1,962* | *155* |
|  |  | **7** | *682* | *463* | *2,260* | *178* |
|  |  | **10** | *766* | *519* | *2,537* | *200* |
|  |  | **14** | *826* | *556* | *2,739* | *217* |
|  | **Required** | **No** | *18* | *13* | *59* | *4* |
|  |  | **1** | *247* | *178* | *817* | *62* |
|  |  | **3** | *382* | *275* | *1,260* | *95* |
|  |  | **5** | *500* | *360* | *1,649* | *124* |
|  |  | **7** | *574* | *414* | *1,895* | *143* |
|  |  | **10** | *638* | *460* | *2,107* | *159* |
|  |  | **14** | *678* | *489* | *2,237* | *169* |
| **0 hours pre-departure** | **Not required** | **No** | *9* | *9* | *29* | *1* |
|  |  | **1** | *279* | *186* | *927* | *75* |
|  |  | **3** | *426* | *288* | *1,411* | *112* |
|  |  | **5** | *558* | *379* | *1,848* | *146* |
|  |  | **7** | *645* | *439* | *2,137* | *168* |
|  |  | **10** | *726* | *492* | *2,406* | *189* |
|  |  | **14** | *785* | *529* | *2,601* | *206* |
|  | **Required** | **No** | *16* | *11* | *52* | *4* |
|  |  | **1** | *228* | *164* | *751* | *57* |
|  |  | **3** | *357* | *258* | *1,180* | *89* |
|  |  | **5** | *472* | *340* | *1,558* | *117* |
|  |  | **7** | *545* | *393* | *1,797* | *136* |
|  |  | **10** | *607* | *438* | *2,004* | *151* |
|  |  | **14** | *646* | *466* | *2,131* | *161* |
| **Entry testing** | **Not required** | **No** | *14* | *13* | *45* | *2* |
|  |  | **1** | *214* | *143* | *709* | *57* |
|  |  | **3** | *359* | *244* | *1,189* | *94* |
|  |  | **5** | *480* | *327* | *1,589* | *125* |
|  |  | **7** | *559* | *382* | *1,853* | *145* |
|  |  | **10** | *633* | *431* | *2,097* | *165* |
|  |  | **14** | *686* | *464* | *2,273* | *179* |
|  | **Required** | **No** | *23* | *16* | *75* | *6* |
|  |  | **1** | *175* | *126* | *576* | *43* |
|  |  | **3** | *304* | *219* | *1,002* | *76* |
|  |  | **5** | *409* | *295* | *1,349* | *102* |
|  |  | **7** | *476* | *343* | *1,570* | *118* |
|  |  | **10** | *533* | *384* | *1,760* | *133* |
|  |  | **14** | *569* | *410* | *1,876* | *142* |

**Table S9** Percentage reduction in missed case (leaked to communities) per 100,000 travelers per week in 336 scenarios of a combination of public health policies for international travelers

| **Testing** | **Vaccination Certificate Requirements** | **Quarantine (Days)** | **Universal** | **Cluster  1** | **Cluster  2** | **Cluster  3** |
| --- | --- | --- | --- | --- | --- | --- |
| **No Testing** | **Not required** | **No** | *** | *** | *** | *** |
| ** Baseline (no interventions)* |  | **1** | *45* | *45* | *45* | *45* |
|  |  | **3** | *58* | *60* | *58* | *57* |
|  |  | **5** | *72* | *74* | *71* | *69* |
|  |  | **7** | *80* | *83* | *80* | *77* |
|  |  | **10** | *88* | *90* | *88* | *85* |
|  |  | **14** | *94* | *95* | *93* | *91* |
|  | **Required** | **No** | *24* | *15* | *25* | *31* |
|  |  | **1** | *58* | *54* | *59* | *63* |
|  |  | **3** | *70* | *67* | *71* | *73* |
|  |  | **5** | *82* | *80* | *82* | *84* |
|  |  | **7** | *89* | *87* | *89* | *90* |
|  |  | **10** | *94* | *94* | *94* | *95* |
|  |  | **14** | *98* | *98* | *98* | *98* |
| **72 hours pre-departure** | **Not required** | **No** | *5* | *4* | *5* | *5* |
|  |  | **1** | *46* | *46* | *46* | *46* |
|  |  | **3** | *59* | *60* | *59* | *58* |
|  |  | **5** | *72* | *74* | *72* | *70* |
|  |  | **7** | *81* | *83* | *81* | *78* |
|  |  | **10** | *88* | *91* | *88* | *86* |
|  |  | **14** | *94* | *96* | *94* | *91* |
|  | **Required** | **No** | *27* | *19* | *27* | *34* |
|  |  | **1** | *59* | *55* | *60* | *63* |
|  |  | **3** | *71* | *68* | *71* | *74* |
|  |  | **5** | *82* | *80* | *82* | *84* |
|  |  | **7** | *89* | *88* | *89* | *90* |
|  |  | **10** | *95* | *94* | *95* | *95* |
|  |  | **14** | *98* | *98* | *98* | *98* |
| **48 hours pre-departure** | **Not required** | **No** | *7* | *6* | *7* | *7* |
|  |  | **1** | *47* | *47* | *47* | *47* |
|  |  | **3** | *60* | *61* | *60* | *59* |
|  |  | **5** | *73* | *74* | *73* | *71* |
|  |  | **7** | *81* | *83* | *81* | *78* |
|  |  | **10** | *89* | *91* | *88* | *86* |
|  |  | **14** | *94* | *96* | *94* | *91* |
|  | **Required** | **No** | *28* | *20* | *29* | *35* |
|  |  | **1** | *60* | *55* | *60* | *64* |
|  |  | **3** | *71* | *68* | *71* | *74* |
|  |  | **5** | *82* | *80* | *82* | *84* |
|  |  | **7** | *89* | *88* | *89* | *90* |
|  |  | **10** | *95* | *94* | *95* | *95* |
|  |  | **14** | *98* | *98* | *98* | *98* |
| **24 hours pre-departure** | **Not required** | **No** | *10* | *9* | *10* | *10* |
|  |  | **1** | *49* | *48* | *49* | *49* |
|  |  | **3** | *61* | *62* | *61* | *60* |
|  |  | **5** | *73* | *75* | *73* | *71* |
|  |  | **7** | *81* | *83* | *81* | *79* |
|  |  | **10** | *89* | *91* | *89* | *86* |
|  |  | **14** | *94* | *96* | *94* | *92* |
|  | **Required** | **No** | *30* | *23* | *31* | *37* |
|  |  | **1** | *61* | *56* | *61* | *65* |
|  |  | **3** | *72* | *69* | *72* | *75* |
|  |  | **5** | *83* | *81* | *83* | *84* |
|  |  | **7** | *89* | *88* | *89* | *90* |
|  |  | **10** | *95* | *94* | *95* | *95* |
|  |  | **14** | *98* | *98* | *98* | *98* |
| **0 hours pre-departure** | **Not required** | **No** | *14* | *13* | *14* | *15* |
|  |  | **1** | *50* | *50* | *50* | *50* |
|  |  | **3** | *62* | *63* | *62* | *61* |
|  |  | **5** | *74* | *76* | *74* | *72* |
|  |  | **7** | *82* | *84* | *82* | *80* |
|  |  | **10** | *89* | *91* | *89* | *87* |
|  |  | **14** | *94* | *96* | *94* | *92* |
|  | **Required** | **No** | *33* | *26* | *34* | *40* |
|  |  | **1** | *62* | *57* | *62* | *66* |
|  |  | **3** | *72* | *69* | *73* | *75* |
|  |  | **5** | *83* | *81* | *83* | *85* |
|  |  | **7** | *89* | *88* | *90* | *90* |
|  |  | **10** | *95* | *94* | *95* | *95* |
|  |  | **14** | *98* | *98* | *98* | *98* |
| **Entry testing** | **Not required** | **No** | *29* | *28* | *29* | *29* |
|  |  | **1** | *53* | *53* | *53* | *54* |
|  |  | **3** | *65* | *66* | *65* | *65* |
|  |  | **5** | *76* | *78* | *76* | *75* |
|  |  | **7** | *84* | *85* | *84* | *82* |
|  |  | **10** | *90* | *92* | *90* | *88* |
|  |  | **14** | *95* | *96* | *95* | *93* |
|  | **Required** | **No** | *45* | *39* | *45* | *50* |
|  |  | **1** | *63* | *59* | *64* | *67* |
|  |  | **3** | *75* | *72* | *75* | *77* |
|  |  | **5** | *84* | *83* | *84* | *86* |
|  |  | **7** | *90* | *89* | *90* | *91* |
|  |  | **10** | *95* | *95* | *95* | *96* |
|  |  | **14** | *98* | *98* | *98* | *98* |

**Table S10** Cluster analysis validation metrics and results

| **Metric** | **Value** |
| --- | --- |
| **Number of Clusters** | 3 |
| **Average Silhouette Width (ASW)** | 0.439 |
| **Calinski-Harabasz Index (CHI)** | 91.62 |
| **Dunn Index (DI)** | 0.052 |
| **Adjusted Ran Index (ARI) : K-means vs Ward's Hierarchical Clustering Method** | 0.782 |
| **Adjusted Ran Index (ARI) : K-means vs Partitioning Around Medoids (PAM)** | 0.829 |

**Table S11** Sensitivity analysis of initial numbers of exposed and infectious compartments

| **Scenarios** | **Initial Numbers of**  ***Exposed% : Infectious%*** | **Numbers of Missed Cases** | **Difference in the Number of Missed Cases Compared to Baseline (%)** | **Reduction of Missed Cases**  **(%)** | **Difference in the Percent Reduction of Missed Cases (%)** |
| --- | --- | --- | --- | --- | --- |
| **No intervention** | **50: 50 (Baseline)** | **863.22** |  |  |  |
|  | *25 : 75* | *854.94* | *8.28 (0.96)* |  |  |
|  | *75 : 25* | *871.49* | *-8.27(-0.95)* |  |  |
| **Minimum intervention** | **50 : 50 (Baseline)** | **821.93** |  |  |  |
|  | *25 : 75* | *810.14* | *-11.79 (-0.01)* |  |  |
|  | *75 : 25* | *833.72* | *11.79 (0.01)* |  |  |
| **Maximum intervention** | **50 : 50 (Baseline)** | **17.55** |  | **98** |  |
|  | *25 : 75* | *17.45* | *0.1 (0.57)* | *98* | *0 (0)* |
|  | *75 : 25* | *17.64* | *-0.09 (-0.51)* | *98* | *0 (0)* |
| **Minimum intervention :** *only 72-hours pre-departure testing* | |  |  |  |  |
| **Maximum intervention :** *72-hours pre-departure testing, vaccination certificate is required, 14-days quarantine, best cluster of departure country based on risk (Cluster 3)* | | | | | |

**Table S12** Sensitivity analysis of false-positive results in susceptible and recovered compartments

**Table S13** Comparison of missed cases estimation: Cluster-specific traveler numbers vs fixed numbers assumption

**Table S14** List of risks identified for imported case reported between April 2020 to April 2022. (AOQ = alternative organizational quarantine; AQ = alternative quarantine; ASQ/ALQ = alternative state quarantine/alternative local quarantine; HQ/AHQ = hotel quarantine/alternative hotel quarantine; OQ = organizational quarantine)

**Table S15-A** List of key PHPITs during the four periods with estimated imported cases for model validation

**Table S15-B** List of key PHPITs during 4 periods with estimated imported case for model validation

**Figure S7** Rate of imported cases per 100,000 travelers per week by case type compared with model data for the same rate within a similar period of combinations of PHPIT options.
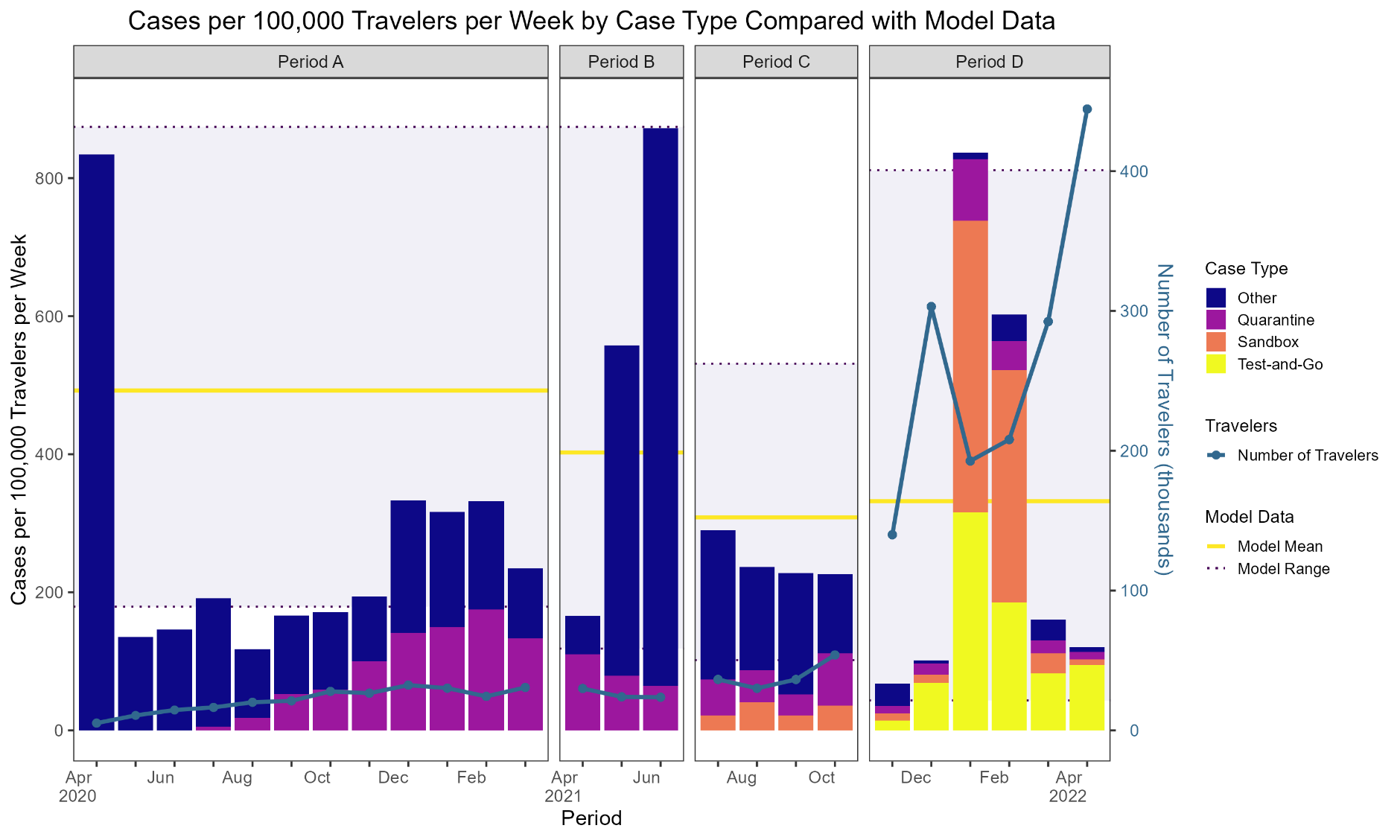
**Figure S8** Rate of imported cases per 100,000 travelers per week by cluster type compared with model data for the same rate within a similar period of combinations of PHPIT options.


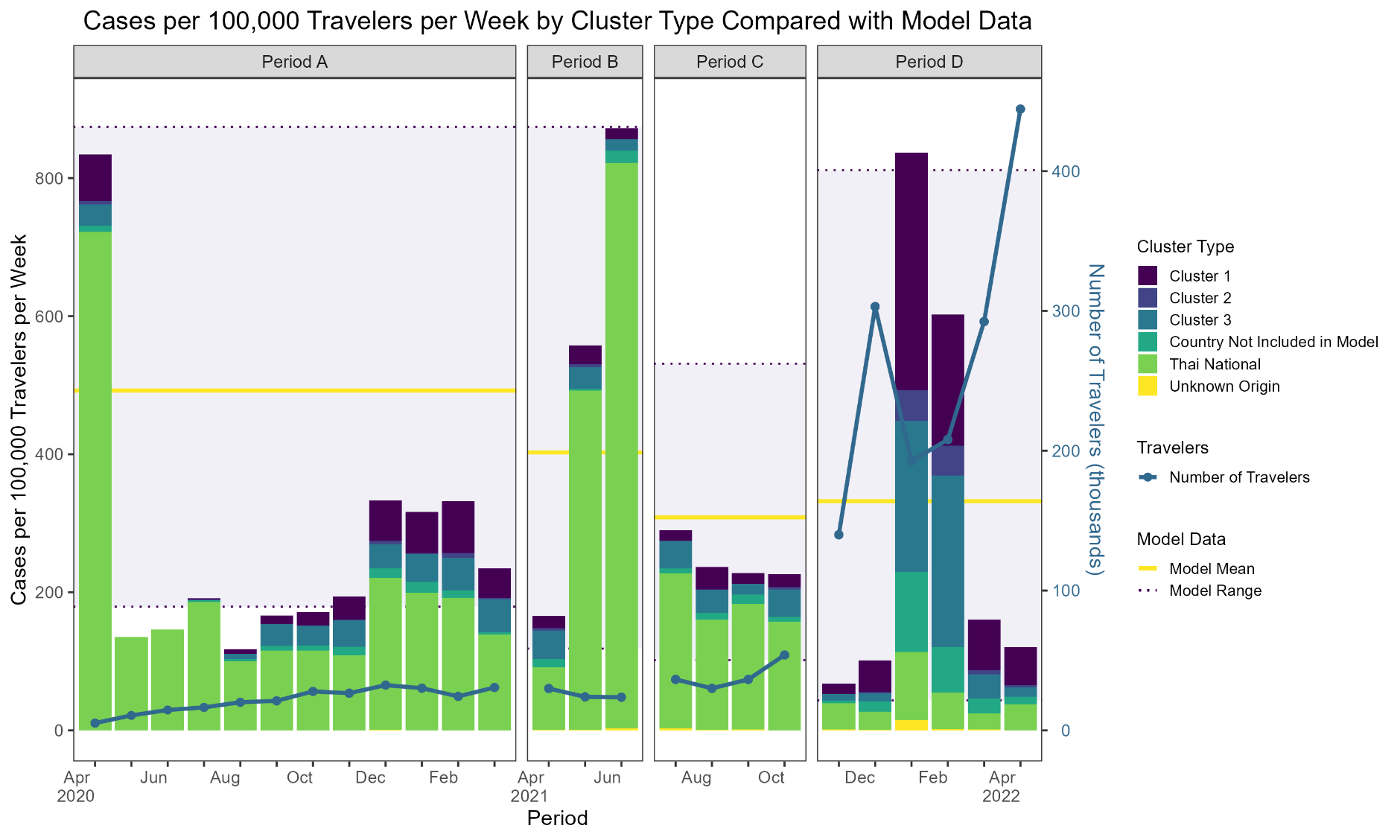


**Reference**

1. WHO. World experts and funders set priorities for COVID-19 research. 2020.

2. Zhou F, Zhou X-H. Confusion about the definition of imported cases in the early stage of the epidemic. Int J Infect Dis. 2021;105:413. https://doi.org/10.1016/j.ijid.2021.02.084.

3. Department of Consular Affairs. Thailand Pass FAQs. Department of Consular Affair, Ministry of Foreign Affais. 2022. https://consular.mfa.go.th/th/content/thailand-pass-faqs-2. Accessed 26 Jan 2023.

4. Cetron M, Maloney S, Koppaka R, Simone P. ISOLATION AND QUARANTINE: CONTAINMENT STRATEGIES FOR SARS 2003. National Academies Press (US); 2004.

5. ECDC. Guidance on quarantine of close contacts to COVID-19 cases and isolation of COVID-19 cases, in the current epidemiological situation, 7 January 2022. European Centre for Disease Prevention and Control. 2022. https://www.ecdc.europa.eu/en/covid-19/prevention-and-control/quarantine-and-isolation. Accessed 22 Jan 2023.

6. WHO. Digital Documentation of COVID-19 Certificates: Vaccination Status. TECHNICAL SPECIFICATIONS AND IMPLEMENTATION GUIDANCE. Green Ink Publishing Services Ltd.; 2021.

7. IATA. Cabin Air & Low Risk of On Board Transmission. 2020. https://www.iata.org/en/youandiata/travelers/health/low-risk-transmission/. Accessed 23 Jan 2023.

8. Aviation Public Health Initiative. Assessment of Risks of SARS-CoV-2 Transmission during Air Travel and Non-Pharmaceutical Interventions to Reduce Risk. Phase One Report: Gate-to-Gate Travel Onboard Aircraft. Harvard T.H. Chan School of Public Health; 2020.

9. Royal Thai Embassy Washington D.C. Thailand Plus, User Manual. https://thaiembdc.org/wp-content/uploads/2021/01/ThailandPlus-Eng.pdf. Accessed 18 Apr 2021.

10. Visa Section Royal Thai Embassy Seoul. Phuket Sandbox Scheme. 2021.

11. Visa section Royal Thai Embassy Seoul. Announcement No.10 on the Entry Procedures for Non-Thai Nationals who are Eligible to Enter the Kingdom of Thailand. 2021.

12. Rome Buathong. 17 TA : Points Of Entry - POEs and Border Health. 2022.

13. Timeline of the COVID-19 pandemic in Thailand. Wikipedia. 2025.

14. Thongmeensuk S, Rojsirikulchai T, Ariyasunthorn A. The Implementation and Phase-out of Travel Bans: A Case Study of Thailand. TDRI; 2022.

15. KPMG Thailand. GMS Flash Alert 2021. 2021.

16. Newsroom TAT. Thailand to lift pretravel testing requirement for international arrivals from 1 April 2022. TAT Newsroom. 2022. https://www.tatnews.org/2022/03/thailand-to-lift-pretravel-testing-requirement-for-international-arrivals-from-1-april-2022/. Accessed 6 Aug 2025.

17. Kucirka LM, Lauer SA, Laeyendecker O, Boon D, Lessler J. Variation in False-Negative Rate of Reverse Transcriptase Polymerase Chain Reaction–Based SARS-CoV-2 Tests by Time Since Exposure. Ann Intern Med. 2020;173:262–7. https://doi.org/10.7326/M20-1495.

18. Swan DA, Bracis C, Janes H, Moore M, Matrajt L, Reeves DB, et al. COVID-19 vaccines that reduce symptoms but do not block infection need higher coverage and faster rollout to achieve population impact. Sci Rep. 2021;11:15531. https://doi.org/10.1038/s41598-021-94719-y.

19. Feikin DR, Higdon MM, Abu-Raddad LJ, Andrews N, Araos R, Goldberg Y, et al. Duration of effectiveness of vaccines against SARS-CoV-2 infection and COVID-19 disease: results of a systematic review and meta-regression. The Lancet. 2022;399:924–44. https://doi.org/10.1016/S0140-6736(22)00152-0.

20. A.  Lauer S, H.  Grantz K, Bi Q, K.  Jones F, Zheng Q, R.  Meredith H, et al. The Incubation Period of Coronavirus Disease 2019 (COVID-19) From Publicly Reported Confirmed Cases: Estimation and Application. Annals of Internal Medicine. 2020. https://doi.org/10.7326/M20-0504.

21. van Kampen JJA, van de Vijver DAMC, Fraaij PLA, Haagmans BL, Lamers MM, Okba N, et al. Duration and key determinants of infectious virus shedding in hospitalized patients with coronavirus disease-2019 (COVID-19). Nat Commun. 2021;12:267. https://doi.org/10.1038/s41467-020-20568-4.

22. WHO. Living guidance for clinical management of COVID-19. 2021.

23. ISO. Country Code. Online Browsing Platform. 2025. https://www.iso.org/obp/ui/#search. Accessed 2 Oct 2025.
